# Supplementary material for: Contribution of amygdala to dynamic model arbitration under uncertainty
Source: Nat Commun. 2025 Nov 28;16:11704. doi: 10.1038/s41467-025-66745-1 (PMC12753643; doi:10.1038/s41467-025-66745-1)
Supplement: Supplementary file 1 — Supplementary Information [file 41467_2025_66745_MOESM1_ESM.pdf]

Supplementary Information for

Contribution of amygdala to dynamic model arbitration under uncertainty

Jae Hyung Woo[1], Vincent D. Costa[2], Craig A. Taswell[3], Kathryn M. Rothenhoefer[2], Bruno B. Averbeck[3], Alireza Soltani[1]

[1]Department of Psychological and Brain Sciences, Dartmouth College, Hanover, NH, USA.
[2]Division of Developmental and Cognitive Neuroscience, Emory National Primate Research Center, Atlanta, GA, USA.
[3]Laboratory of Neuropsychology, National Institute of Mental Health, National Institutes of Health, Bethesda, MD, USA

Correspondence should be addressed to Alireza Soltani (alireza.soltani@dartmouth.edu)

Supplementary Note 1

Considering previous findings on faster decision making during action-based compared with stimulus-based tasks[24], we hypothesized that reaction time (RT) on a given trial depends on the learning system that controls the behavior more strongly on that trial. To test this hypothesis, we categorized trials as either stimulus- or action-dominant by directly comparing $ERDS_{Stim}$ and $ERDS_{Action}$ (computed from a moving window of ten trials). Trials that had $ERDS_{Stim} < ERDS_{Action}$ were categorized as stimulus-dominant, and action-dominant if $ERDS_{Action} < ERDS_{Stim}$. To assess whether the dominant strategy had a significant effect on RT, we performed mixed-effects analysis and identified the unique effect of the dominant strategy from other potential confounds. Specifically, the mixed-effects model included subject-level random intercepts and random slopes for the following fixed effects predictors: dominant strategy, coded as stim-dominant (0) or action-dominant (1), whether the monkey has chosen the better option (1) or not (0), reward schedule or uncertainty, trial number within a block, block number within a session, and session number within the subject. We also included interaction between dominant strategy and choice of better option, as the latter could depend on the adopted strategy. Variables were normalized for each monkey to yield comparable standardized regression coefficients.

For the What-only task in control monkeys, the vast majority of the analyzed trials (79.4%) were classified as stimulus-dominant (**Supplementary Figure 1a**). Interestingly, there was still a proportion of trials with $ERDS_{Action} < ERDS_{Stim}$ (8.66% ±1.25%) even though action-based value/strategy was irrelevant for performing the task. Therefore, although the What-only task lacks the task-imposed, objective uncertainty about the correct model of the environment, monkeys still considered this uncertainty and the alternative model of the environment. The mixed-effects analysis indicated that the dominant strategy was not a significant predictor of RT ($\beta_{Act\text{-}Dominant} = 0.0111$, $p = .866$; M±SEM across subjects for stimulus-dominant RT: 223.8±29.5; action-dominant RT: 225.2±34.9). To compare the performance for these two types of trials, we ran a similar generalized mixed-effects model predicting animals' choice of better option (binomial distribution with logit link function), with the same random and fixed effects. Using this analysis, we found that action-dominant trials predicted significantly lower performance ($\beta_{Act\text{-}Dominant} = -0.708$, $p = 3.54\times10^{-238}$), as reflected in the *P*(*Better*) of action-dominant trials (0.621±0.010) and stimulus-dominant (0.790±0.016) trials. These results suggest that most action-dominant trials happened when action values were used instead of stimulus values to make decisions, resulting in more erroneous responses.

For the What/Where task, the proportions of stimulus-dominant and action-dominant trials were reflected in the respective block types: the What blocks were marked by a higher proportion of stimulus-dominant trials (59.5%±5.99%; **Supplementary Figure 1b**), whereas the majority of trials within the Where blocks were categorized as action-dominant (63.6%±6.41%; **Supplementary Figure 1c**). Critically, performance was higher for the correct strategy: in the What blocks, performance was significantly higher for stimulus-dominant (0.820±0.035) than action-dominant (0.607±0.019) trials ($\beta_{Act\text{-}Dominant} = -0.959$, $p = 4.94\times10^{-324}$), whereas in the Where blocks, *P*(*Better*) was higher for action-dominant (0.791±0.025) than for stimulus-dominant (0.597±0.018) trials ($\beta_{Act\text{-}Dominant} = 0.846$, $p = 4.94\times10^{-324}$). In terms of RT, we found that for both block types, action-dominant strategy predicted significantly shorter RT ($\beta_{Act\text{-}Dominant}$

= -0.119, p = 2.14×10^{-13}). Consistently, categorization of trials based on comparison of ERDS (stimulus-dominant vs. action-dominant) yielded a larger distinction in RTs than categorization simply based on block type (What vs. Where), as reflected by the smaller proportion of variance explained by block type predictor (R^2 equal to 0.005 and 0.0081 for block type and dominant strategy; comparison of partial R^2 values). These results show that entropy-based metrics could be used to identify the adopted model on a given trial and that RT reflected the adopted strategy, with the stimulus-based strategy yielding consistently longer RT than the action-based strategy. This could be explained by the fact that the monkeys can prepare the left or right movement early on to make decisions based on actions, whereas a stimulus-based decision requires the animals to identify the target and then plan the movement only after the stimuli appear. We note that the results remain qualitatively the same when the analyses are restricted to the last 20 trials of each block, where performance has plateaued (**Supplementary Figure 2**).

In brain-lesioned monkeys, the effect of the dominant strategy on RT was more consistent across two tasks, with the action-dominant strategy significantly predicting shorter RTs (**Supplementary Figure 1d–i**). Furthermore, the correct strategy for a given block type predicted significantly higher performance. That is, action-dominant strategy predicted lower performance during the What-only task (amygdala: $\beta_{\text{Act-dominant}}$ = -0.230, p = 1.35×10^{-63}; VS: $\beta_{\text{Act-dominant}}$ = -0.459, p = 1.65×10^{-143}) and What blocks of What/Where task (amygdala: $\beta_{\text{Act-dominant}}$ = -0.665, p = 8.26×10^{-308}; VS: $\beta_{\text{Act-dominant}}$ = -0.320, p = 701×10^{-52}), whereas it predicted higher performance in the Where blocks of What/Where task (amygdala: $\beta_{\text{Act-dominant}}$ = 0.546, p = 1.01×10^{-152}; VS: $\beta_{\text{Act-dominant}}$ = 0.550, p = 1.93×10^{-101}).

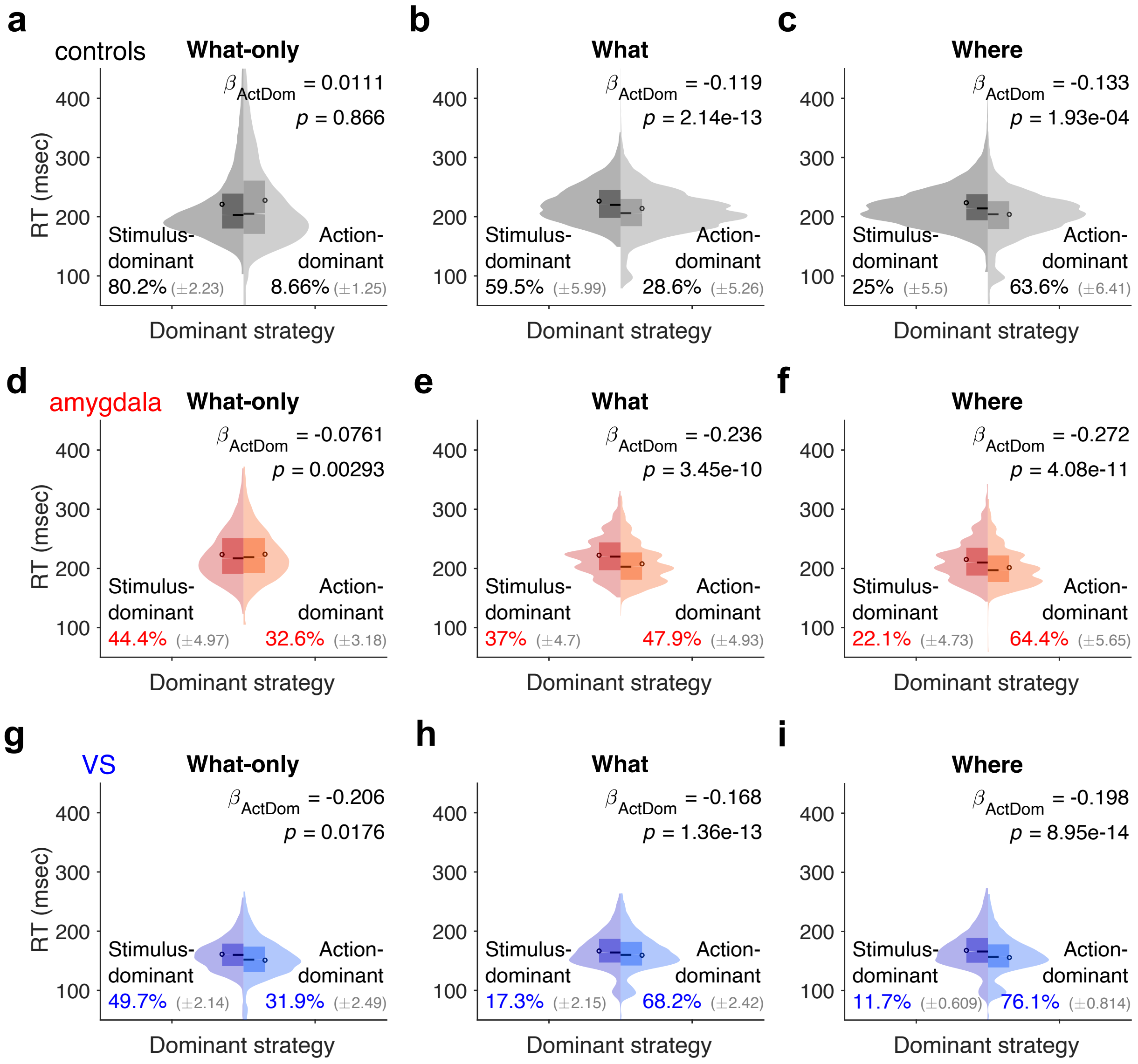

Supplementary Figure 1. Distributions of reaction time (RT) for stimulus-dominant and action-dominant trials, separately for different tasks and block types. Each trial was categorized as either stimulus- or action-dominant by comparing $ERDS_{Stim}$ and $ERDS_{Action}$. Percentages indicate proportions of trials under each category, reported as mean ± SEM across subjects (remaining percentages correspond to trials where both strategies were equally dominant). (**a–c**) RT data from control monkeys during the What-only (a) and the What/Where tasks (b, c). n=133520 (What-only), n=240560 (What), n=234960 trials (Where). (**d–f**) RT data from amygdala-lesioned monkeys during the What-only (d) and the What/Where tasks (e, f). n=122720 (What-only), n=78800 (What), n=70480 blocks (Where). (**g–i**) RT data from VS-lesioned monkeys during the What-only (g) and the What/Where tasks (h, i). n=71920 (What-only), n=67920 (What), n=67600 blocks (Where).Circles in the violin plots represent means and black horizontal lines represent medians of the distributions. Reported are standardized regression coefficient and corresponding p-values for the main effect of action-dominant strategy, with negative (positive) values indicating that action-dominant trials predict shorter (longer) RT. In the What/Where task (b, c, e, f, h, i), RT was consistently shorter for action-dominant trials across all groups. Source data are provided as a Source Data file.

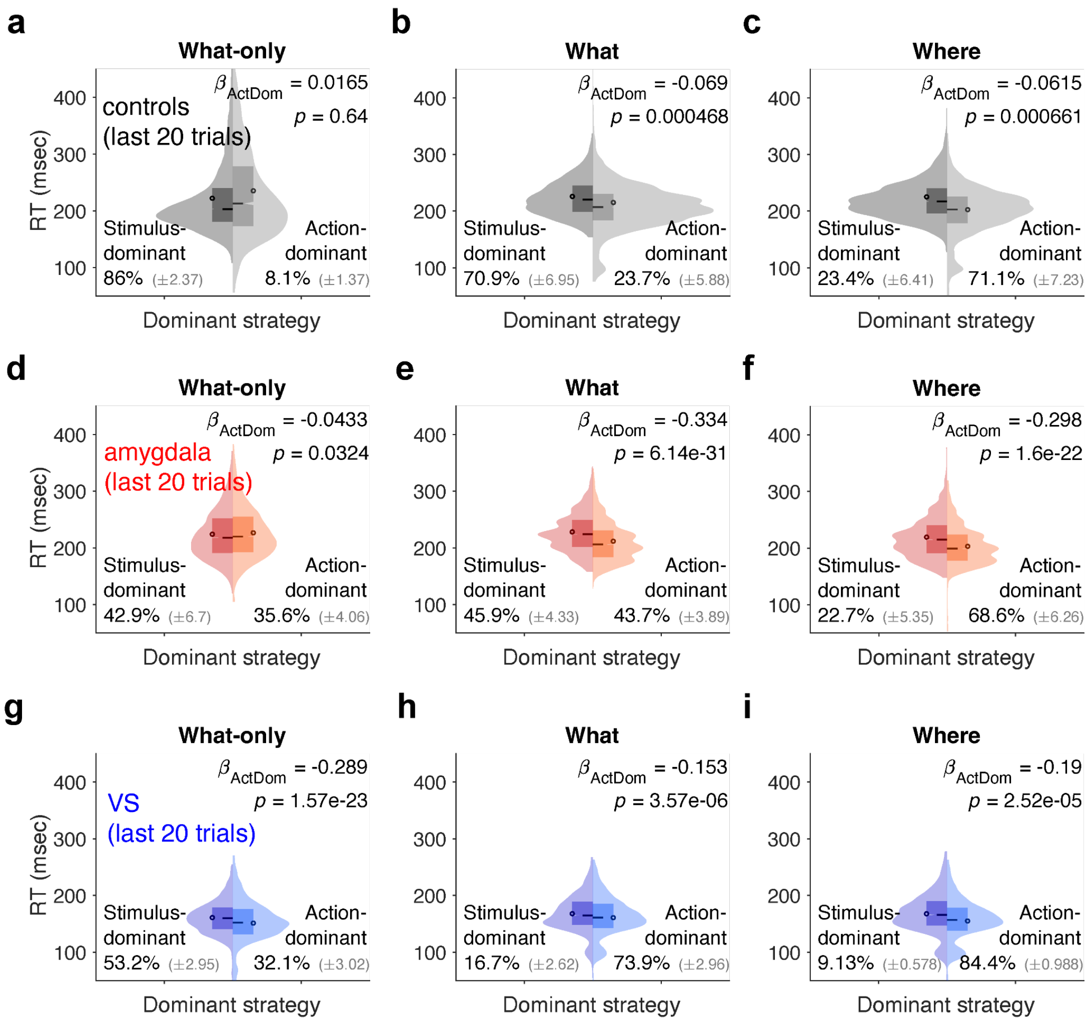

Supplementary Figure 2. Distributions of reaction time (RT) for stimulus-dominant and action-dominant trials, restricted to the last portion of the block. Same analysis as in **Supplementary Figure 1**, but restricted to the last 20 trials (last 25%) of each block where performance has reached a plateau. Reported are standardized regression coefficient and corresponding p-values for the main effect of action-dominant strategy. (**a–c**) RT data from control monkeys: n=32525 (What-only), n=60123 (What), n=58692 (Where) trials. (**d–f**) RT data from amygdala-lesioned monkeys: n=29740 (What-only), n=19666 (What), n=17596 (Where) trials. (**g–i**) RT data from VS-lesioned monkeys: n=16636 (What-only), n=16758 (What), n=16661 (Where) trials. Source data are provided as a Source Data file.

Supplementary Note 2

In **Supplementary Note 1**, we showed that the more dominantly adopted strategy has a significant effect on RT, with a stimulus-dominant strategy requiring longer RT regardless of the given block type. Based on these findings, we also tested whether the predictors derived from the RL model (Dynamic ω-ρ) yield consistent results. That is, we hypothesized that the RL model's arbitration weight Ω, quantifying the relative weight of stimulus- and action-based system in driving choice, is a significant modulator of RT. This quantity provides a more fine-grained measure of relative strategy compared to the ERDS-based approach used in **Supplementary Note 1**, which binarizes the otherwise continuous degree of relative strategy into action- or stimulus-dominant. To this end, we first confirmed that the dynamic Ω values estimated by the winning model (Dynamic ω-ρ) better captures the RT data than the Static ω model that assumes lack of adjustment in Ω, across all groups and tasks (**Supplementary Figure 3**). Based on these results, we then used a mixed-effects analysis with subject-level random intercepts and random slopes for the following fixed effects predictors: arbitration weight (Ω), absolute overall value difference ($|\Delta OV| = |OV_{Left} - OV_{Right}|$), whether the animal's choice was the "correct" option (in terms of higher reward probability), trial number within a block, block number within a session, and session number within the subject. Note that in this analysis, the block type (for the What/Where task) and reward uncertainty were not included as predictors, because these were objective variables unknown to the monkeys and were instead captured by arbitration weight and the value difference (reflecting monkey's subjective estimates).

Our analysis based on model-derived estimates (**Supplementary Figure 4**) showed overall positive effects of arbitration weight on RT across all tasks and groups, supporting the view that action-based decision is faster than stimulus-based decision. During the What-only task, the regression weights of arbitration weight for control and amygdala-lesioned monkeys were positive although not significant (controls: β_Ω = 0.0113, p = .632; **Supplementary Figure 4a**; amygdala: β_Ω = 0.0536, p = .149; **Supplementary Figure 4c**), while VS-lesioned monkeys showed significant effect (β_Ω = 0.162, $p = 5.27\times10^{-3}$; **Supplementary Figure 4e**). This could be related to the fact that arbitration weights in control monkeys were saturated toward the stimulus-based system in this task (see **Figure 2d**), in contrast to the lesioned groups that exhibited more mixture of two strategies (e.g., **Figure 3b, c**). In particular, the largest proportion of variance in VS group's RT was accounted by the arbitration weight ($R^2_\Omega = 3.97\times10^{-5}$, followed by $R^2_{Block\#} = 4.16\times10^{-6}$ and $R^2_{|\Delta OV|} = 7.27\times10^{-7}$; partial R^2 values), suggesting its significant role in reducing RT of VS-lesioned monkeys. Furthermore, across all groups, we found significant effects of |ΔOV|, such that the larger distinction between two choice options led to significantly shorter RT (controls: $\beta_{|\Delta OV|}$ = -0.0824, p = .00909; amygdala: $\beta_{|\Delta OV|}$ = -0.064, $p = 1.45\times10^{-4}$; VS: $\beta_{|\Delta OV|}$ = -0.118, $p = 3.88\times10^{-11}$). These results illustrate the RL model's ability to capture the animals' subjective value estimates and demonstrate that the observed effects of the arbitration weight on RT are unique from other included effects.

During the What/Where task, which required more explicit arbitration between two strategies, all groups showed significant effects of Ω on RT, with higher Ω predicting slower RT (controls: β_Ω = 0.117, $p = 4.63\times10^{-5}$; amygdala: β_Ω = 0.161, $p = 4.30\times10^{-12}$; VS: β_Ω = 0.0475, p = .0273). These results further support the claim that the relative dominance of the strategy, even when

measured in a continuous scale, can significantly predict the animals' RT. Of particular relevance to the VS lesion is the significant effect of arbitration weight on RT: in the What-only task, VS group seems to show a large modulatory effect of Ω on RT, such that a mixture of action-based strategy results in the significantly reduced RT compared to the other groups. In the What/Where task, the effect of Ω on RT is relatively moderate compared to the first task, yet since VS group heavily relies on action-based strategy for this task (small Ω), the resulting RT is also predicted to be smaller. Overall, these results point to the potential role of arbitration in the hastening RT due to VS lesions.

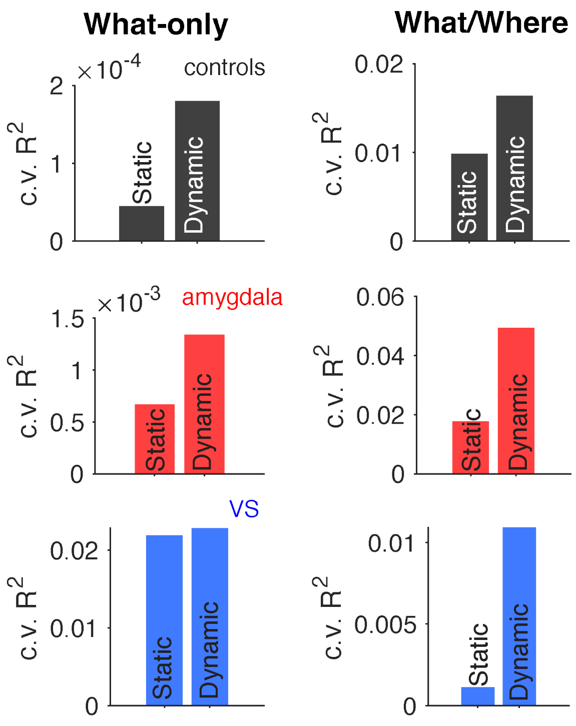

Supplementary Figure 3. Comparison of static and dynamic arbitration weights in predicting RT data. Plotted are mean 10-fold cross-validated R-squared measures of regression models predicting RT with static or dynamic arbitration weight, inferred by respective models. To directly compare between two types of arbitration weight, we used regression models with just a single predictor (either static or dynamic arbitration weight). The dynamic arbitration weight outperforms static arbitration weight in predicting RT, as suggested by higher R^2 in held-out trials. This is consistent with the model fitting results, which have shown that dynamic arbitration weight is superior in predicting choice behavior compared to the static models. Source data are provided as a Source Data file.

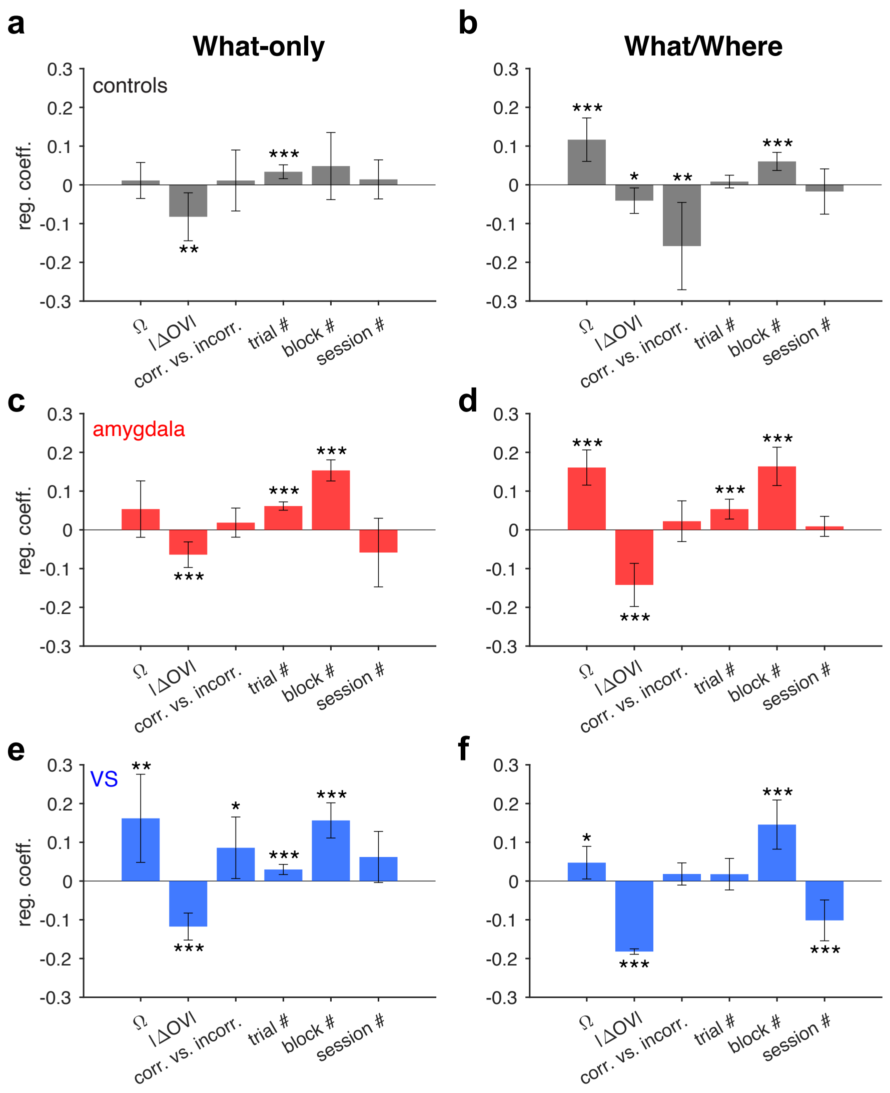

Supplementary Figure 4. Regression of reaction time (RT) on arbitration weight and other task-related variables. Plotted are standardized regression coefficients from a mixed-effects model with subjects as a random effect, for given lesioned groups and tasks. Asterisk indicates significant effect of a given predictor (*: $p < .05$, **: $p < .01$, ***: $p < .001$). *corr. vs. incorr.*: the main effect of choosing the option with higher reward probability (correct option). (**a, c, e**) Regression coefficients for predicting RT data during What-only task, in controls (a; n=130099 trials), amygdala- (c; n=119460), and VS-lesioned (e; n=66402) monkeys. (**b, d, f**) Regression coefficients for predicting RT data during What/Where task, in controls (b; n=475209 trials), amygdala- (d; n=149001), and VS-lesioned (f; n=133618) monkeys. Overall, task-related variables have similar impacts on RT in all groups. Source data are provided as a Source Data file.

Supplementary Note 3

Our model fitting relies on maximum likelihood estimation (MLE), a method that maximizes the likelihood function ($\mathcal{L}(\theta_i;\boldsymbol{y})= P(\boldsymbol{y}|\theta_i)$), ensuring that the observed data ($\boldsymbol{y}$) is the most likely outcome under the model defined by the parameters (θ_i). In our case, the data consist of the animals' choices on each trial of the experiment ($C(t)$, where to $C(t)$ = 1 and 0 for left and right choices), leading to the optimization of the following expression for each block of 80 trials:

$$\ell = \ln \mathcal{L}(\theta_i;\boldsymbol{y}) = \ln\left[P(\boldsymbol{y}|\theta_i)\right] = \ln\left[\prod_{t=1}^{80}\left\{P_{Left}(t)\,C(t) + (1-P_{Left}(t))(1-C(t))\right\}\right] \qquad (Eq.\,\mathrm{S1})$$

where $P_{Left}(t)$ is the probability of choosing left as predicted by the model (see Eq. 5 in the Methods), and the logarithm of the likelihood function ($\ell = ln\,\mathcal{L}$, referred to as the log-likelihood) is used to perform the optimization more conveniently.

We then used cross-validated negative log-likelihoods ($-LL$) of the alternative models to identify the best-fitting model, where a smaller negative LL indicates a better fit. Notably, even a small improvement in negative LL reflects a significant increase in the likelihood that the superior model better explains the observed choice behavior. To illustrate this, consider an example comparing the negative log-likelihoods of two models, i and j ($-LL_i$ and $-LL_j$):

$$\Delta LL = -LL_i - (-LL_j) \qquad (Eq.\,\mathrm{S2})$$

where LL_i is the mean log-likelihood of model i across the N test blocks:

$$LL_i = \frac{1}{N}\sum_{b=1}^{N}\ell(y_b|\theta_i) \qquad (\mathrm{Eq.\,S3})$$

where $\ell(y_b|\theta_i)$ represents the optimized log-likelihood of the model i for the data in block b (y_b), as described in Eq. S1 above. Noting that all blocks consist of 80 trials, it then follows:

$$\Delta LL = \frac{1}{N}\sum_{b=1}^{N}\left(\ell(y_b|\theta_j) - \ell(y_b|\theta_i)\right) = \frac{1}{N}\sum_{b=1}^{N}\left\{\ln\left[\frac{P(y_b|\theta_j)}{P(y_b|\theta_i)}\right]\right\}, \qquad (\mathrm{Eq.\,S4})$$

and therefore,

$$N \times \Delta LL = \sum_{b=1}^{N}\left\{\ln\left[\frac{P(y_b|\theta_j)}{P(y_b|\theta_i)}\right]\right\} = \ln\left[\prod_{b=1}^{N}\frac{P(y_b|\theta_j)}{P(y_b|\theta_i)}\right]. \qquad (\mathrm{Eq.\,S5})$$

By taking exponential on both sides, we obtain

$$e^{N\times\Delta LL} = \frac{\prod_b P(y_b|\theta_j)}{\prod_b P(y_b|\theta_i)}, \qquad (\mathrm{Eq.\,S6})$$

which leads to the relationship between the likelihoods of the two models:

$$\prod_{b}^{N} P(y_b|\theta_j) = e^{N\times\Delta LL} \times \prod_{b}^{N} P(y_b|\theta_i)\,. \qquad \text{(Eq. S7)}$$

Considering that the median number of tested blocks across subjects for a given cross-validation instance was 92 (M±SD = 97.11±30.6, with a minimum of 48, corresponding to 20% of the blocks), even a slight difference in the log-likelihood, such as ΔLL = 0.05 (indicating the superiority of Model *j*), implies that the test data is approximately $e^{92*0.05} = 99.48$ times more likely to have originated from Model *j* compared to Model *i* on average.

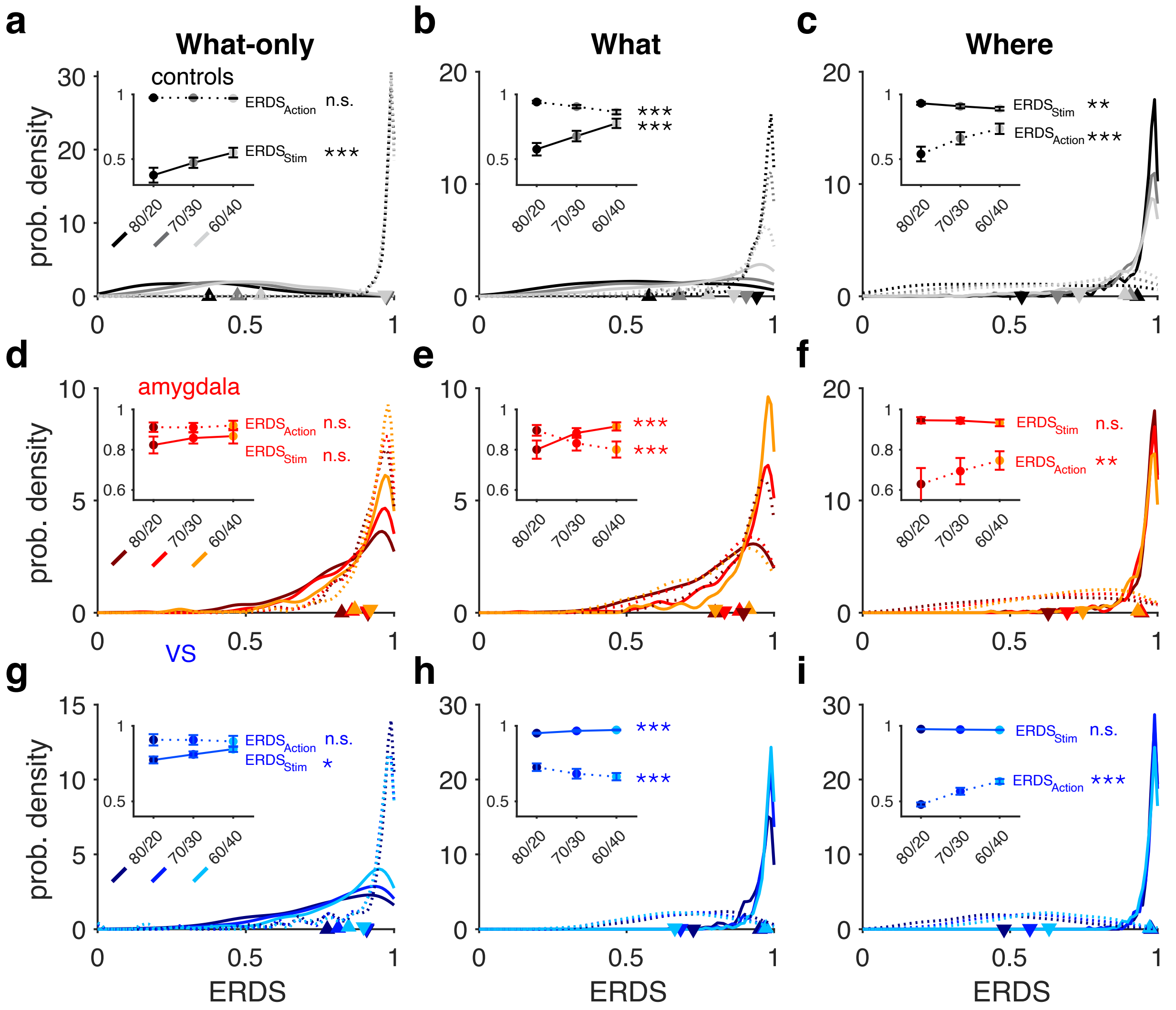

Supplementary Figure 5. Distributions of conditional entropy of reward-dependent strategy (ERDS) by block type and reward schedule. Distributions of the ERDS based on stimulus identity ($ERDS_{Stim}$, in solid lines) or action ($ERDS_{Action}$, in solid lines) computed from each of 80-trial blocks during What-only (first column) and What/Where task (second and third columns). Insets represent mean values (Error bars = SEM across subjects). Asterisks indicate significant effect of reward uncertainty on ERDS, using mixed-effects analysis with subjects as a random effect (*: p < .05, **: p < .01, ***: p < .001), with the following model specifications: What-only: ERDS ~ *reward_var* + (1+*reward_var*+*sess_perc*+*block_in_sess* |subject); What/Where: ERDS ~ *reward_var*blockType* + (1+*reward_var*blockType* +*sess_perc*+*block_in_sess* |subject). Reward uncertainty of each block was measured as the variance of outcome[13], i.e., p_{Better}*(1– p_{Better}). (**a–c**) $ERDS_{Stim}$ (in solid lines) and $ERDS_{Action}$ (in dotted lines) in control monkeys during What-only (a) and What/Where tasks (b, c). Colors indicate reward schedules (black: 80/20; dark gray: 70/30, light gray: 60/40). n=1669 (What-only), n=5944 (What/Where). (**d–f**) ERDS in amygdala-lesioned during What-only (d) and What/Where tasks (e, f). Colors indicate reward schedules (brown: 80/20; red: 70/30, orange: 60/40). n=1534 (What-only), n=1866 (What/Where). (**g–i**) ERDS in VS-lesioned during What-only (g) and What/Where tasks (h, i). Colors indicate reward schedules (navy: 80/20; blue: 70/30, cyan: 60/40). n=899 (What-only), n=1694 (What/Where). Source data are provided as a Source Data file.

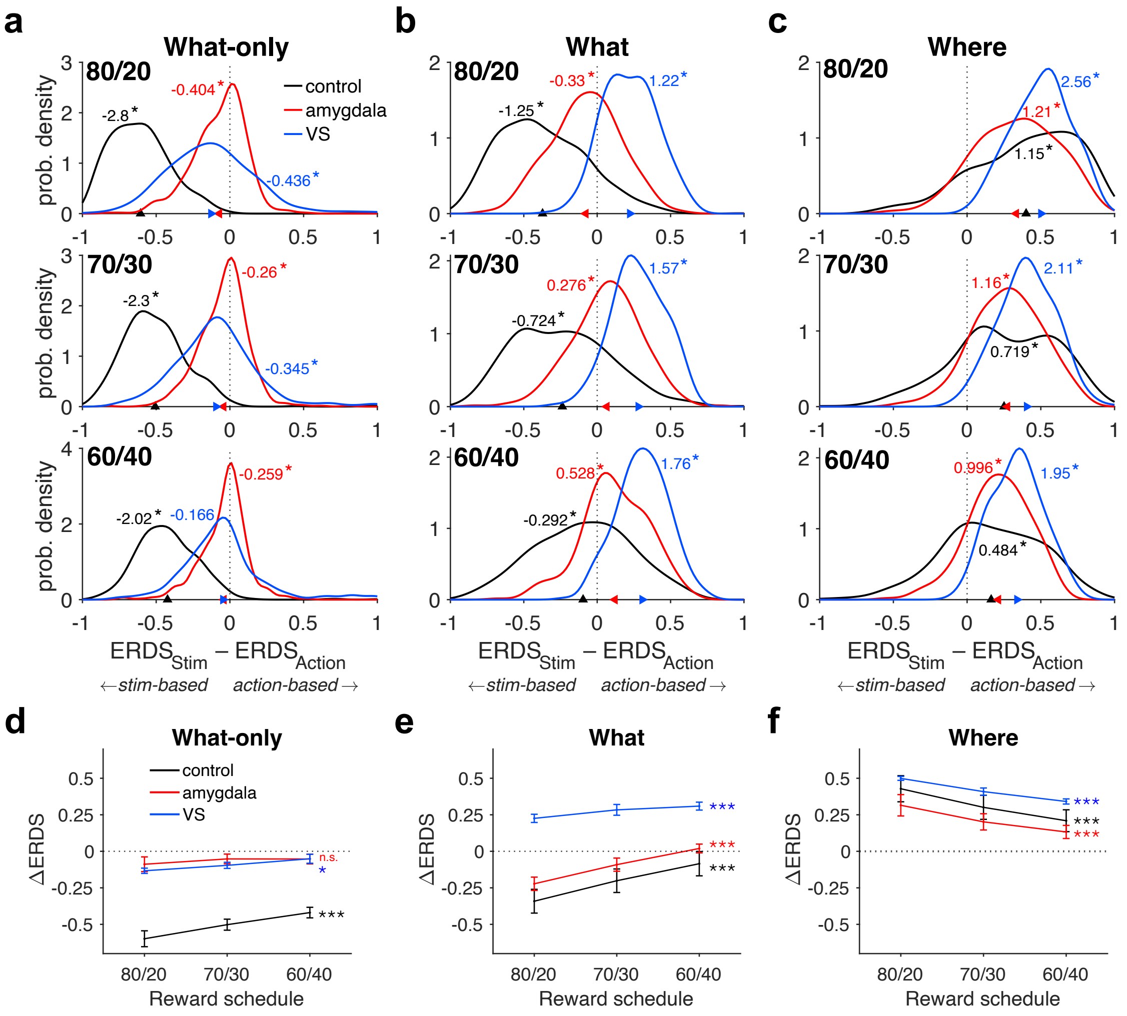

Supplementary Figure 6. Interactions between stimulus-based and action-based learning depend on the uncertainty of the reward environment. (**a–c**) Distributions of the difference between ERDS based on stimulus identity and action ($ERDS_{Stim} – ERDS_{Action}$) computed from each of 80-trial blocks, separately for different reward schedules during What-only (a) and What/Where task (b, c). Reported are Cohen's *d* values from paired-samples t-test between two metrics, and asterisks next to the value indicate significance ($p < .001$). Colors indicate control (black), amygdala-lesion (red), and VS-lesion (blue) monkeys. Triangles on the X-axis indicate means of the difference for each group. Negative $ERDS_{Stim} – ERDS_{Action}$ values indicate dominance of the stimulus-based strategy, whereas positive values indicate dominance of the action-based strategy. In controls, means (black triangles) and Cohen's *d* values become closer to zero as reward schedules are more uncertain. What-only: n=551/555/563 (controls, for each reward schedule, respectively), n=505/520/509 (amygdala), n=310/309/280 (VS); What: n=1020/985/1001 (controls); n=325/314/346 (amygdala); n=287/279/283 (VS); Where: n=981/984/972 (controls); n=307/297/277 (amygdala); n=281/288/276 (VS). (**d–f**) Summary results for the panels in a–c. Plotted are mean values of $\Delta ERDS = ERDS_{Stim} – ERDS_{Action}$, by each group and reward schedule during What-only (d) and What/Where (e,f) tasks. With larger reward uncertainty, animals' strategies became relatively more biased toward the incorrect strategy (increasing ΔERDS in the What blocks and decreasing ΔERDS in the Where block). Asterisks next to the plots indicate significant effect of reward uncertainty (variance) for the respective group indicated by colors, as determined by mixed-effects analysis (same models as in **Supplementary Figure 5**; *: $p < .05$, **: $p < .01$, ***: $p < .001$). It is worth noting that, while $ERDS_{Action}$ was not explicitly modulated by reward uncertainty during the What-only task for any of the groups (**Supplementary Figure 5a, d, g**), competitive interactions between $ERDS_{Stim}$ and $ERDS_{Action}$ still exist for the task. This suggests that $ERDS_{Stim}$ cannot be solely responsible for the observed effect in ΔERDS. Source data are provided as a Source Data file.

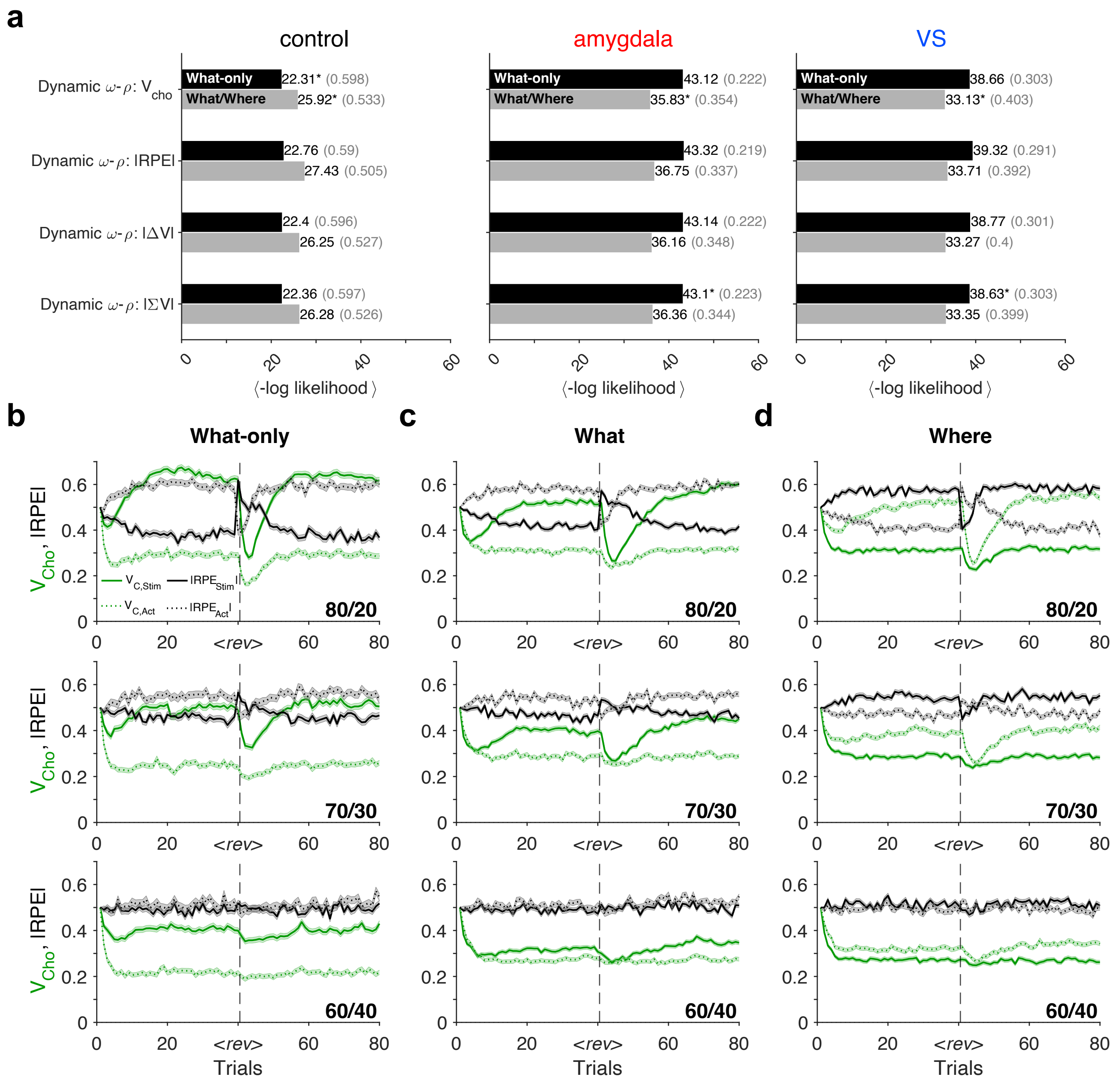

Supplementary Figure 7. Comparison of different reliability signals for dynamic arbitration between the stimulus- and action-based systems. (**a**) Goodness of fit using five-fold cross-validation of the Dynamic ω-ρ model with different types of reliability signals. V_{cho}: value of chosen option V_{cho}, |RPE|: magnitude of RPE, |ΔV|: magnitude of difference between chosen and unchosen options, ΣV: sum of chosen and unchosen options. Numbers in parenthesis indicate McFadden R^2. Reliability signal based on V_{cho} consistently yields the best fit to choice behavior across all block types in control animals. (**b–d**) V_{cho} signal provides better separability between reliable and unreliable systems. Plotted are control monkeys' averaged trajectories for the value of the chosen option (V_{cho}, green) and magnitude of RPE (|RPE|, black) within each system (stimulus-based for solid, and action-based for dotted lines) as a function of trial number during What-only (b), What (c), and Where (d) blocks. Each row corresponds to each reward schedule: Top = 80/20, middle = 70/30, bottom = 60/40. In these plots, we used estimated parameters from the Static ω model ($RL_{Stim+Action}$ +Static ω) to avoid confounds in signals in dynamic models. Source data are provided as a Source Data file.

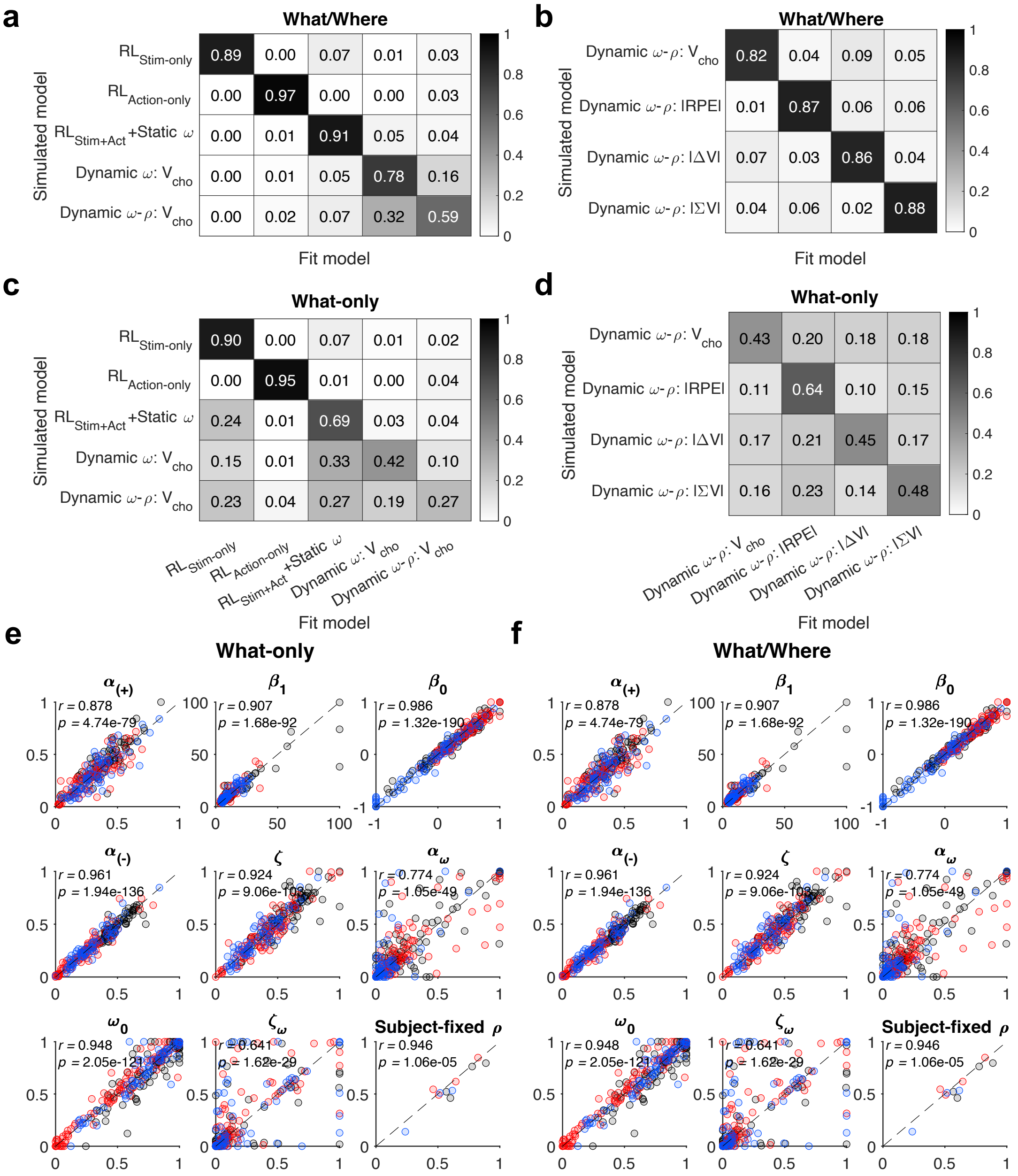

Supplementary Figure 8. Models and parameters recovery. (**a**) Model recovery of one- and two-system models, with and without dynamic arbitration. Reported numbers are proportion of sessions that are best explained by a given fitted model (minimum AIC) for each simulated model. The Dynamic ω-ρ model is less distinguished from the Dynamic ω model, as the latter is a special case of the former model with ρ = 0.5. (**b**) Model recovery for alternative reliability signals in dynamic arbitration, as shown in **Supplementary Figure 7**. (**c, d**) Same plot as in a and b, but for the What-only task. In this task, simulated behaviors of more complex models become less distinct from their simpler counterparts. (**e, f**) Scatter plots of true (X-axis) and recovered (Y-axis) parameters from the Dynamic ω-ρ model. Each panel shows each parameter of the model for the three groups of monkeys (controls: black; amygdala-lesioned: red; VS-lesioned: blue). Reported are Pearson's correlation between true and recovered parameters. n=243 (What-only), n=532 sessions (What/Where). All correlations were $r > 0.60$ and significant ($p < .001$). See **Methods** and **Supplementary Table 15** for detailed description of the fitted parameters. ρ is assumed to be fixed for each monkey, whereas all other parameters are estimated for each session. Source data are provided as a Source Data file.

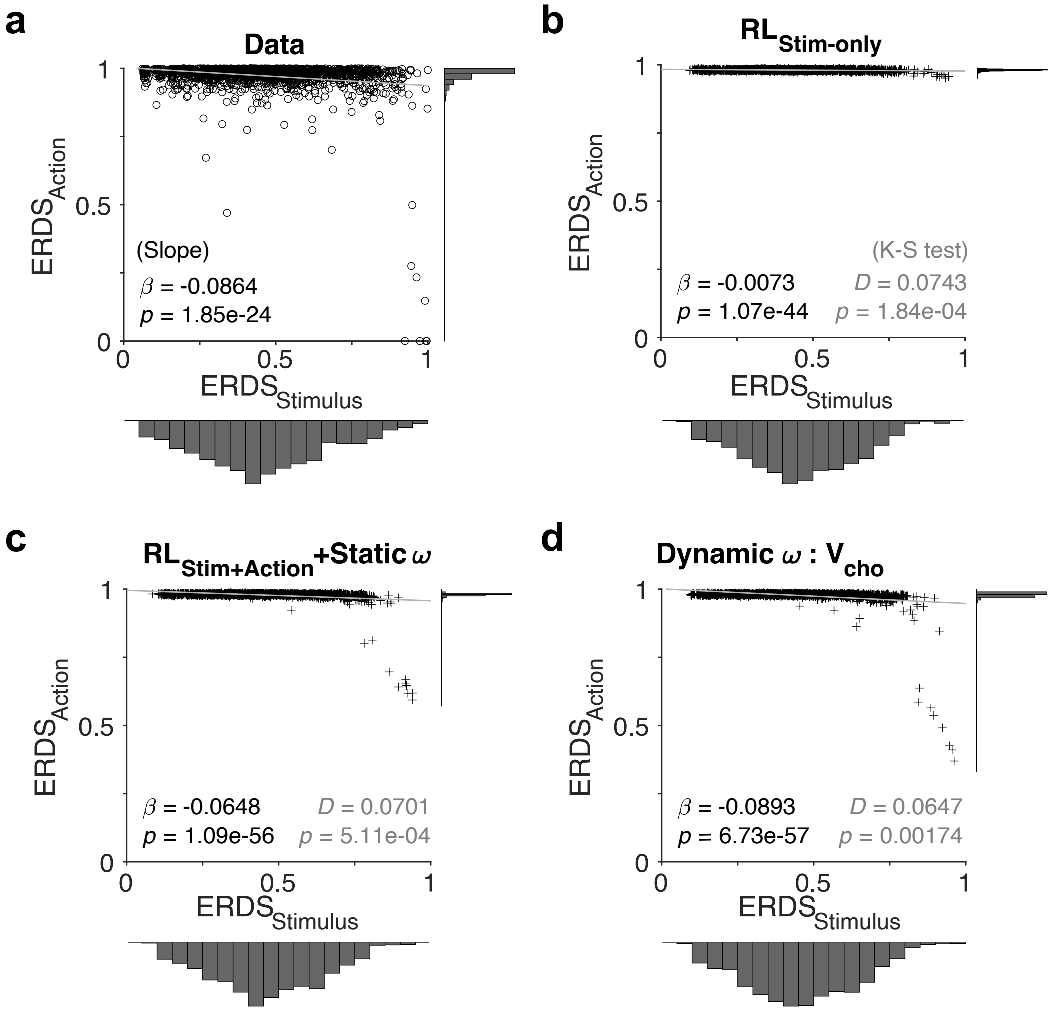

Supplementary Figure 9. Comparison of empirical and simulated ERDS in control monkeys during the What-only task provides evidence for presence of multiple learning systems. (**a**) Scatter plot of entropy of reward-dependent strategy based on stimulus identity ($ERDS_{Stim}$, X-axis) vs. action ($ERDS_{Action}$,Y-axis) in control monkeys during the What-only task. Reported are regression coefficient (β) and its p-value for the regression of $ERDS_{Action}$ on $ERDS_{Stim}$, using mixed-effects analysis with subjects as a random effect ($ERDS_{Action}$ ~ $ERDS_{Stim}$ + (1|subject), mean-centered predictors by subject; n=1669 blocks). Histograms in the X- and Y-axis show distributions of $ERDS_{Stim}$ and $ERDS_{Action}$, respectively. (**b**) Simulated metrics using estimated parameters of the model with stimulus-learning system only ($RL_{Stim-only}$). Reported in bottom right is the test statistics (D-values) and its p-value from the two-sample Kolmogorov-Smirnov test, comparing the distance between the distributions of ΔERDS = $ERDS_{Stim}$ – $ERDS_{Action}$ from the data (a) and the simulated model. (**c**) Simulated metrics using estimated parameters of the static two-system model ($RL_{Stim+Action}$+Static ω). (**d**) Simulated metrics using estimated parameters of the two-system model with dynamic adjustment using V_{chosen} as the reliability measure (Dynamic ω: V_{cho}). The Dynamic ω model better captures the variability in $ERDS_{Action}$ compared to the Static ω model shown in panel (c), as reflected in the more similar regression coefficient (β) to the data and the smaller distance to the empirical distribution of ΔERDS (D-values). Source data are provided as a Source Data file.

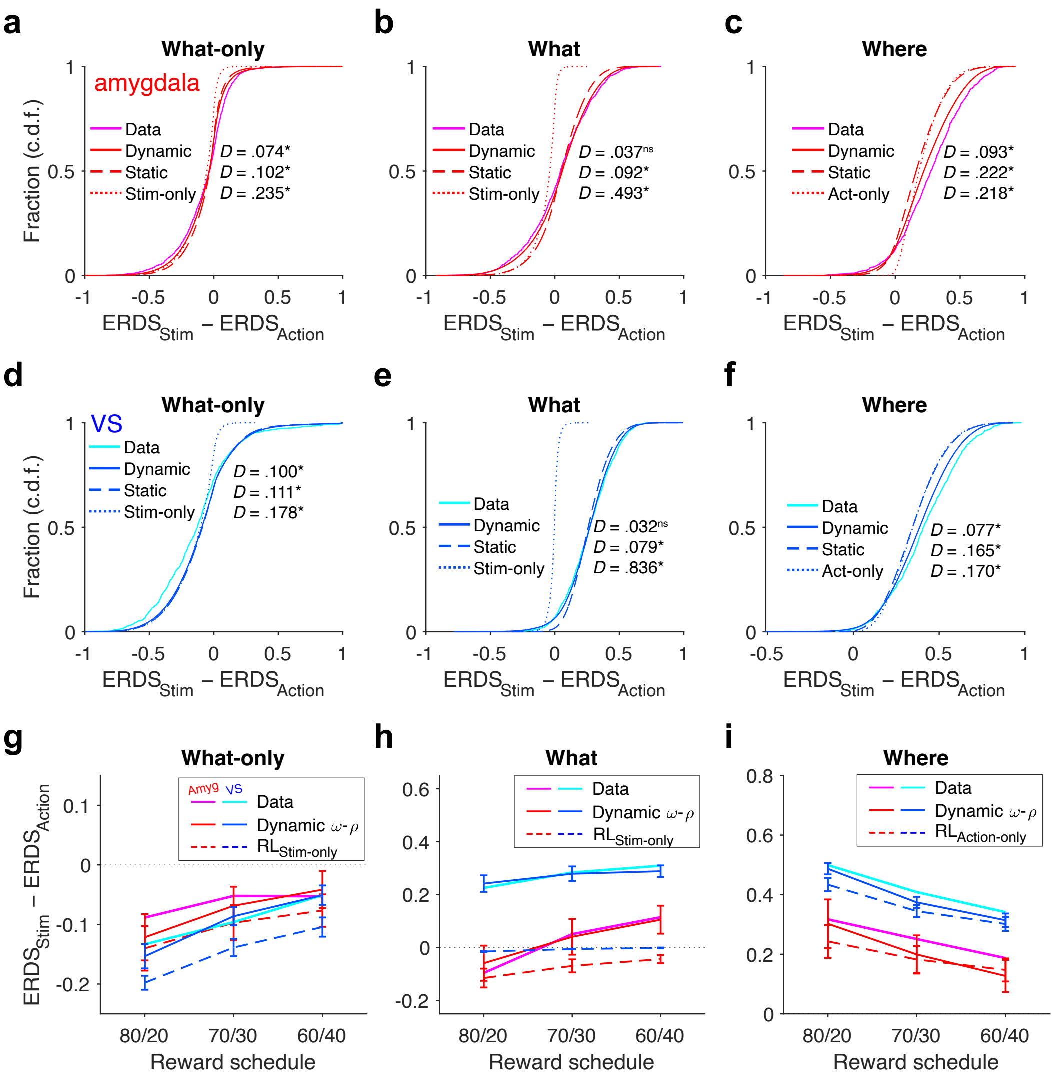

Supplementary Figure 10. Model validation results demonstrate that the model with dynamic arbitration better captures the relative strength of the two strategies compared to the model with no arbitration. (**a**) Plotted are cumulative distribution functions (CDF) of empirical (magenta) and simulated (red) values of the relative strength of two strategies ($ERDS_{Stim}$ – $ERDS_{Action}$ during each block) in the amygdala-lesioned monkeys. Dynamic ω-ρ, Static ω, and one-system (stimulus-only) models are compared. Reported values next to each model are the test statistics (D-values) using the two-sample Kolmogorov-Smirnov test, comparing the distance between the distributions of data and the indicated model. Asterisks next to the D-values indicate significance ($p < .001$; ns = not significant). Asterisks between the model statistics indicate significant differences between the distributions of two models. Each block was simulated 100 times with the fitted parameters of each model (n=153400). (**b**) CDF of empirical and simulated values of $ERDS_{Stim}$ – $ERDS_{Action}$ for the What blocks during the What/Where task (n=98500). Conventions are the same as in (a). (**c**) Same plot as in (b) but for the Where blocks during the What/Where task (n=88100). Action-only model was simulated for the one-system model. (**d–f**) Same plots as in panels **a–c** but for VS-lesioned monkeys (n=89900, 84900, 84500 respectively). (**g–i**) Mean values of $ERDS_{Stim}$ – $ERDS_{Action}$ shown in (a–f), broken down by reward schedules (magenta/cyan lines for empirical data, same as those shown in **Supplementary Figure 6d–f**, and red/blue lines for model simulations), during the What-only (g) and the What/Where task (h, i). Error bars = SEM across subjects. The two-system model with dynamic arbitration, the Dynamic ω-ρ model, more effectively captures the data compared to the single-system models without arbitration. Source data are provided as a Source Data file.

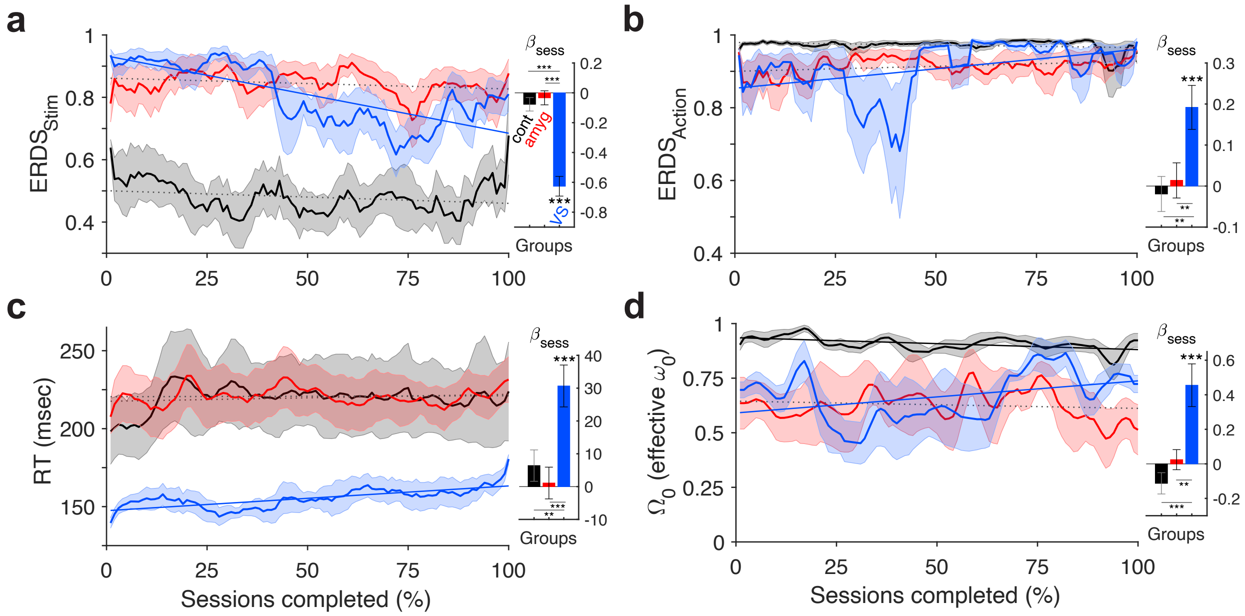

Supplementary Figure 11. Contribution of the amygdala to behavioral adjustments over long timescales. (**a, b**) Time course of entropy of reward-dependent strategy on stimulus identity ($ERDS_{Stim}$, a) and performed action ($ERDS_{Action}$, b) for controls (black), amygdala- (red), and VS-lesioned (blue) monkeys during the What-only task. Number of blocks completed was normalized by each monkey into percentages (error bars indicate SEM across subjects). Straight lines indicate least-squares lines regressing ERDS on the fraction of sessions completed. Sub-panels to the right show regression coefficients for the proportion of sessions completed for each group (corresponding to the slope of the fitted lines but accounting for subject variability). Asterisks indicate significance of coefficient from mixed-effects analyses (n=4102 blocks). Asterisks between bar plots indicate significant group difference in slopes (*: $p < .05$, **: $p < .01$, ***: $p < .001$). Significant effects shown only. Full statistics are available in **Supplementary Table 11–14**. (**c, d**) Time course of median RT (c) and initial effective arbitration weight Ω_0 (d) within each block for the entirety of the What-only task. Conventions are the same as in panels a and b. Note Ω_0 are estimated by sessions (n=243). Source data are provided as a Source Data file.

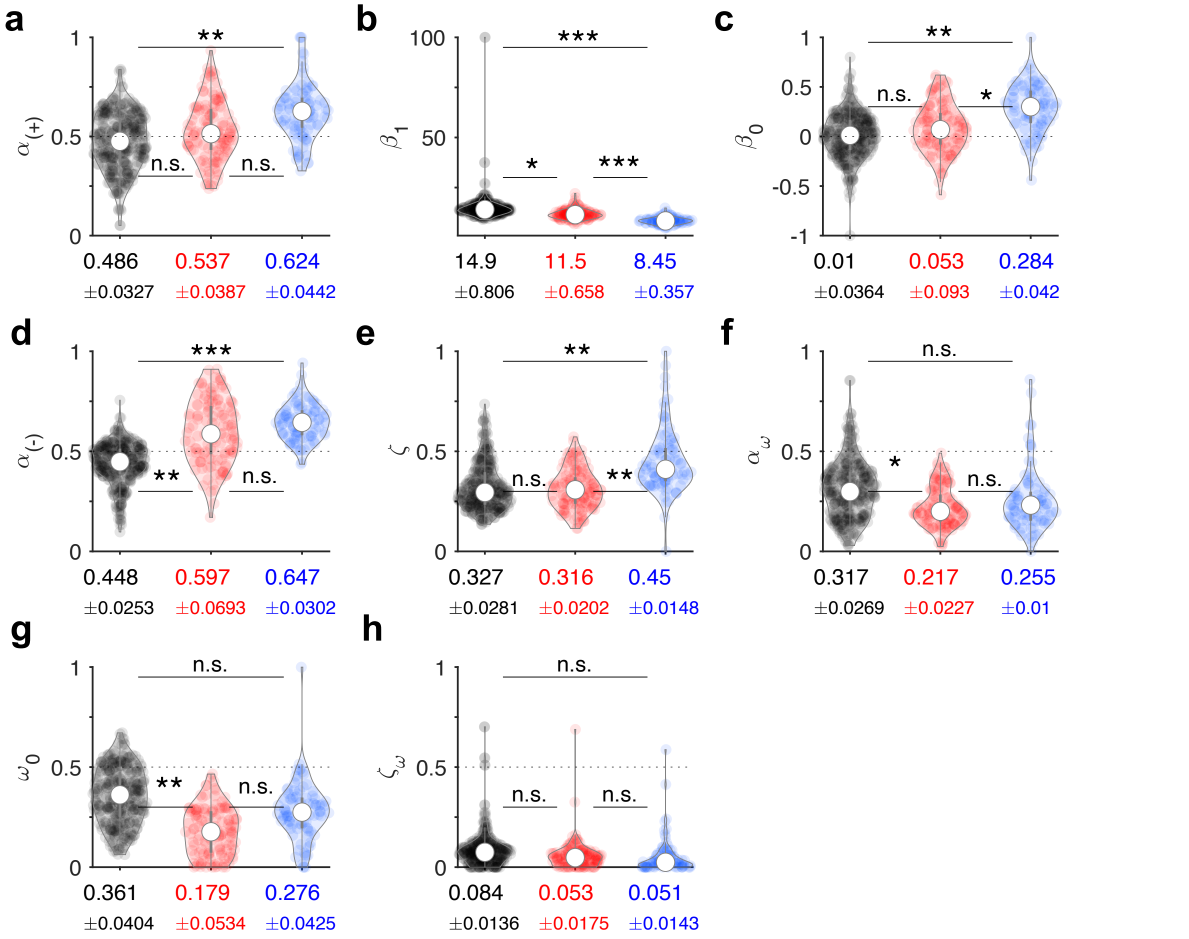

Supplementary Figure 12. Distributions of estimated model parameters in the What/Where task across the three groups. Plotted are the distributions of estimated parameters of the best model (Dynamic ω-ρ with V_{cho}) fitted to the choice behaviors of controls (black), amygdala- (red), and VS-lesioned (blue) monkeys during the What/Where task. α_+: learning rate on rewarded trials (a). β_1: common inverse temperature for stimulus- and action-based systems (b). β_0: side bias term, positive if preferring right option (c). α_-: learning rate on unrewarded trials (d) ζ: decay or forgetting rate for the unchosen option (e). α_ω: arbitration transition rate (f). ω_0: initial arbitration weight on the first trial of each block (g). ζ_ω: decay rate for arbitration weight ω toward initial value (h). Asterisks indicate significant effects of group difference (mixed-effects analysis with contrasts, *parameter* ~ *group* + (1+*sess_perc*|*subject*)); *: $p < .05$, **: $p < .01$, ***: $p < .001$; n=532 sessions). Circles in the violin plots indicate medians, and the numbers on the X-axis indicate mean±SEM. across subjects. ρ parameter is assumed to be fixed for each subject and is shown in **Figure 4a**. Source data are provided as a Source Data file.

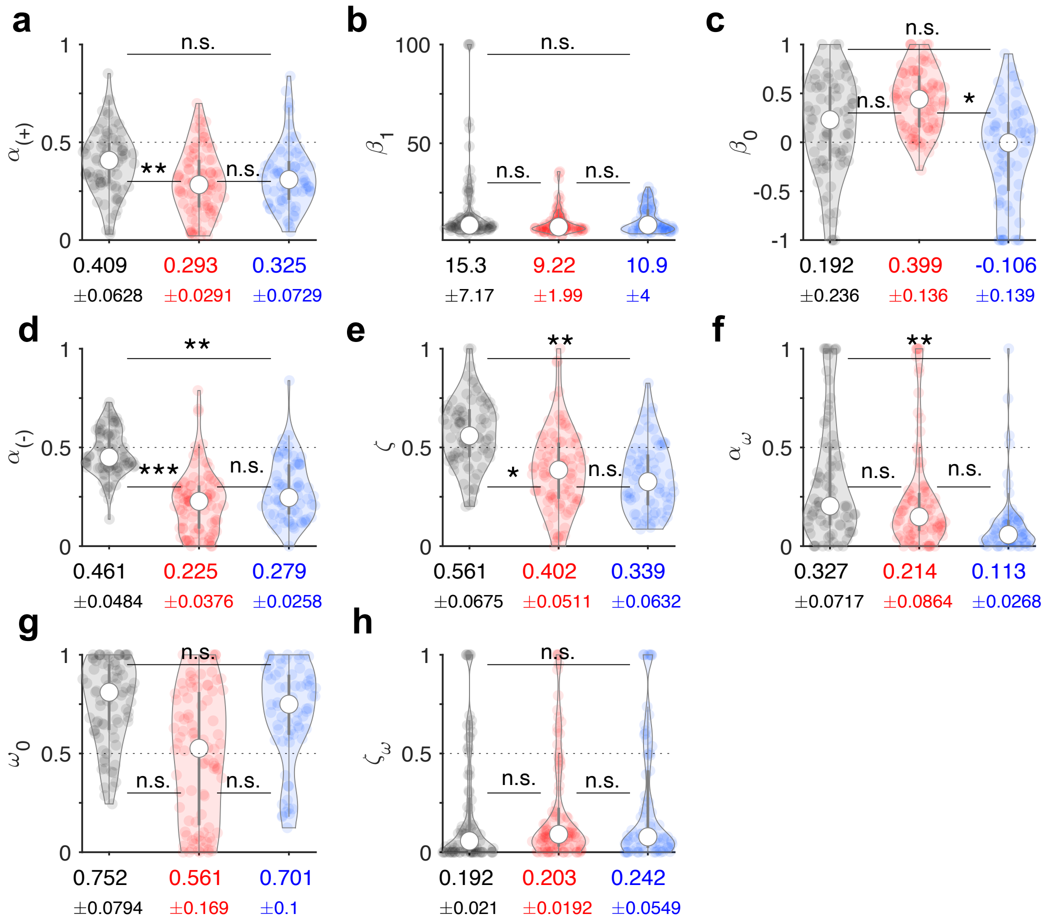

Supplementary Figure 13. Distributions of estimated model parameters in the What-only task across the three animal groups. Plotted are the distributions of estimated parameters of the best model (Dynamic ω-ρ with V_{cho}) fitted to the choice behaviors of controls (black), amygdala- (red), and VS-lesioned (blue) monkeys during the What-only task. α_{+}: learning rate on rewarded trials (a). β_1: common inverse temperature for stimulus- and action-based systems (b). β_0 : side bias term, positive if preferring right option (c). α_{-}: learning rate on unrewarded trials (d) ζ: decay or forgetting rate for the unchosen option (e). α_{ω}: arbitration transition rate (f). ω_0: initial arbitration weight on the first trial of each block (g). ζ_{ω}: decay rate for arbitration weight ω toward initial value (h). Asterisks indicate significant effects of group difference (mixed-effects analysis with contrasts, *parameter* ~ *group* + (1+*sess_perc*|*subject*); *: $p < .05$, **: $p < .01$, ***: $p < .001$; n=243 sessions). Circles in the violin plots indicate medians, and the numbers on the X-axis indicate mean±SEM across subjects. ρ parameter is assumed to be fixed for each subject and is shown in **Figure 4a**. Source data are provided as a Source Data file.

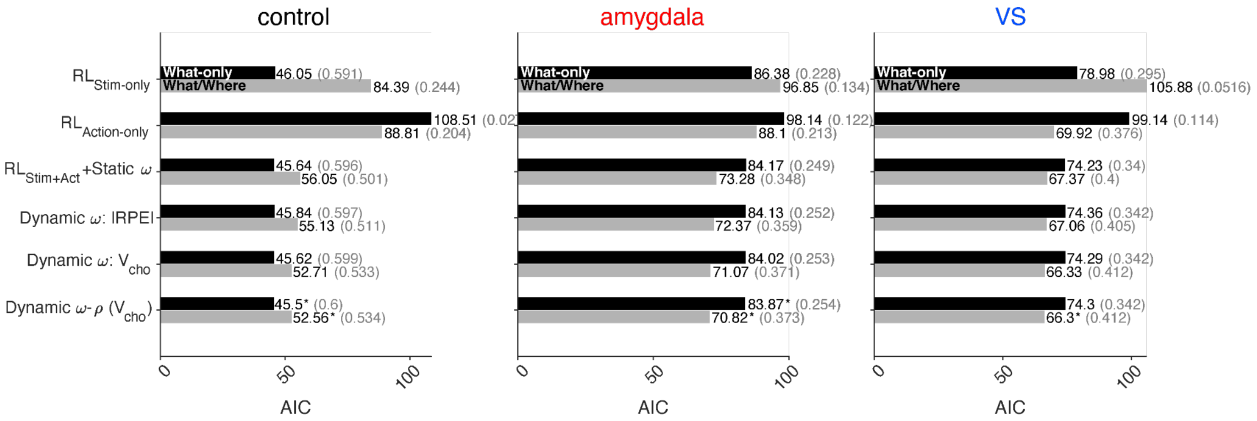

Supplementary Figure 14. Mean AIC of the six models used to fit data in the three groups of monkeys. Plotted are averaged AIC of each fitted session, normalized by block counts within sessions. In all cases, the Dynamic ω-ρ model provides the best fit, consistent with the fitting based on cross-validated negative log-likelihood. Asterisks indicate significant difference between the best and second-best model based on one-sided paired-samples t-test (*: $p < .05$; control: What-only: $p = 4.46 \times 10^{-5}$; What/Where: $p = 3.89 \times 10^{-20}$; Amygdala: What-only: $p = 4.49 \times 10^{-7}$; What/Where: $p = 1.62 \times 10^{-15}$; VS: What/Where: $p = .0393$). Source data are provided as a Source Data file.

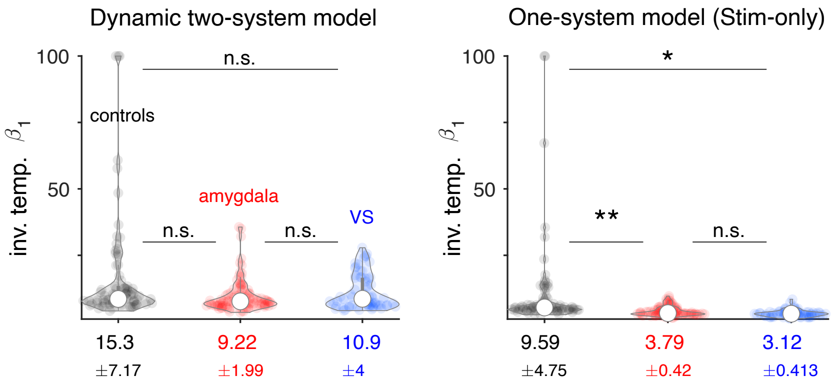

Supplementary Figure 15. Comparison of inverse temperature estimated by two models during What-only task. Plotted are distributions of inverse temperature for each group, estimated from either two-system model with arbitration (left; same as **Supplementary Figure 13b**) or one-system, stimulus-only model (right; similar to Costa et al., 2016) during What-only task. Asterisks indicate significant effects of group difference (mixed-effects analysis with random effects of subjects; *: p < .05, **: p < .01, ***: p < .001, two-sided; n=243 sessions). According to the dynamic two-system model examined in our study, both lesioned groups were associated with overall smaller yet non-significant reduction in inverse temperature compared to controls in the What-only task (mixed-effects analysis; β_{amyg} = -13.57, p = .0581; β_{VS} = -11.56, p = .169). This contrasts with the single-system (Stim-only) model which exhibits more pronounced reduction in the inverse temperature (β_{amyg} = -13.9, p = .00546; β_{VS} = -14.68, p = .0103). This indicates that one-system models make less precise predictions on the value difference and consequently result in the reduced choice consistency to compensate for the lack of precision. Source data are provided as a Source Data file.

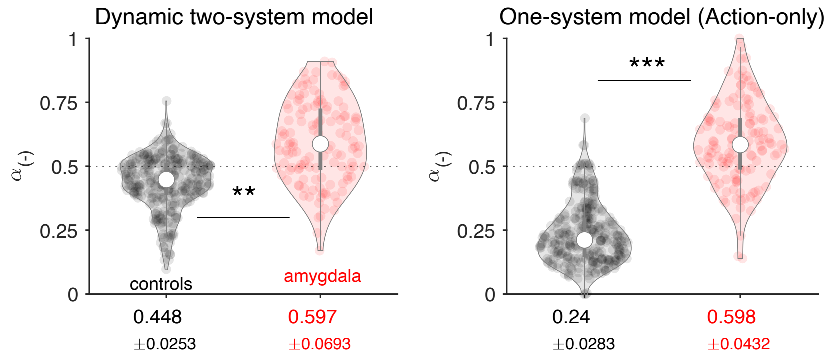

Supplementary Figure 16. Comparison of negative learning rate estimated by two different models during What/Where task. Plotted are distributions of negative learning rates for control and amygdala-lesioned groups, estimated from either two-system model with arbitration (left; same as **Supplementary Figure 12d**) and one-system, action-only model (right; similar to Taswell et al., 2021) during What/Where task. Asterisks indicate significant effects of group difference (mixed-effects analysis with random effects of subjects; *: $p < .05$, **: $p < .01$, ***: $p < .001$, two-sided; n=442 sessions). Increased sensitivity to negative feedback (α-) in amygdala-lesioned monkeys is relatively well preserved across both models. That is, larger α- in amygdala group compared to controls during the What/Where task is reflected in both the dynamic arbitration model (β_{amyg} = 0.169, p = .00895) and the single-system Action-only model (β_{amyg} = 0.367, $p = 5.91\times10^{-10}$; corresponding to the previous study's finding). These results reflect the fact that the sensitivity to reward feedback is a more local aspect of behavior which can be sufficiently explained by single-system models without invoking more complex arbitration components. Source data are provided as a Source Data file.

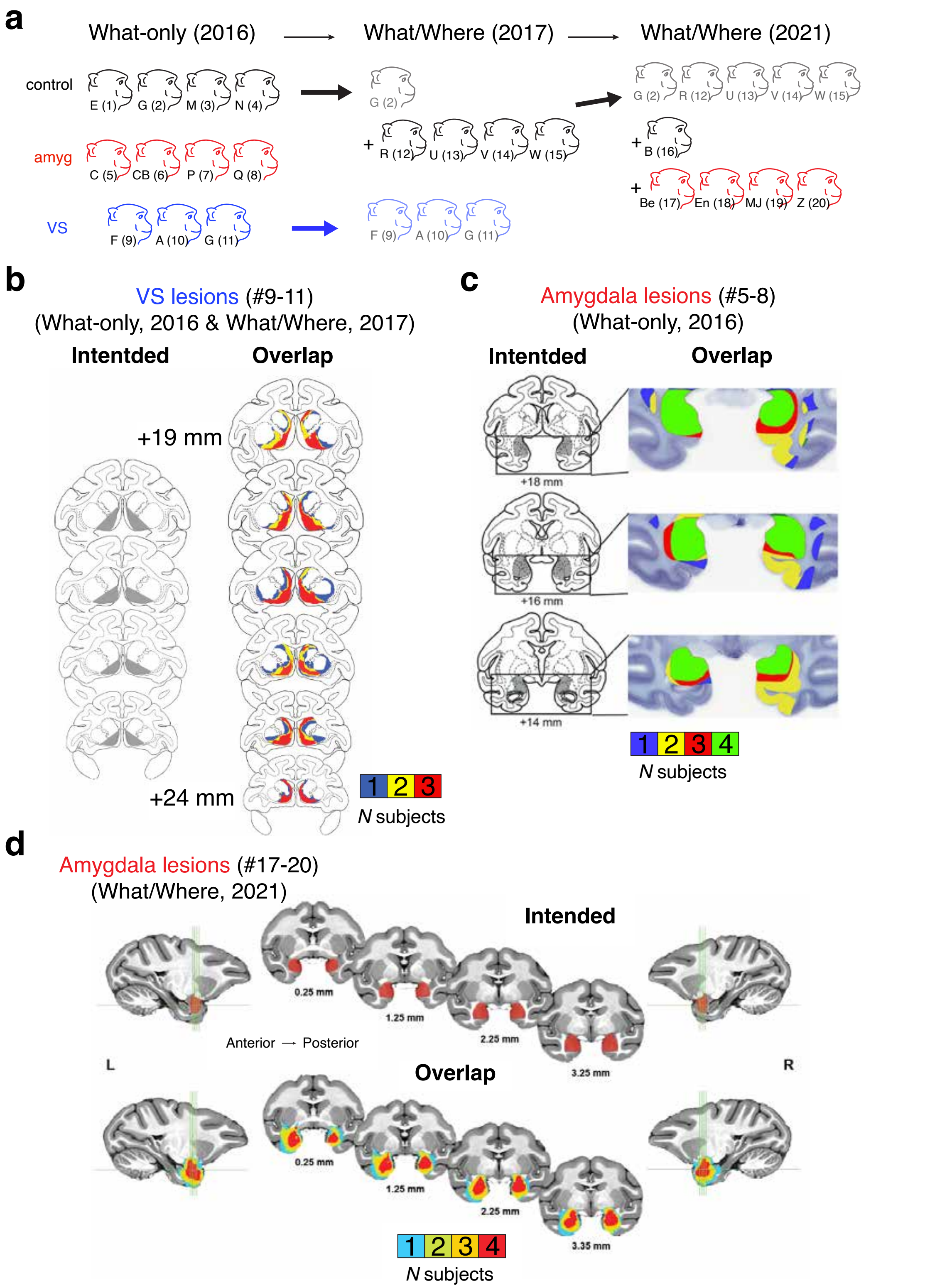

Supplementary Figure 17. Summary of monkeys used in the current study, with references to the original datasets, and the lesion extents mapped for animals with bilateral excitotoxic lesions to amygdala (amyg) or ventral striatum (VS). (**a**) A diagram summarizing all twenty monkeys used for the experiments. Colors indicate lesion groups (black: control; red: amygdala-lesioned; blue: VS-lesioned).

The initial and number on each monkey indicate a unique identifier for each subject. The plus sign (+) indicates new monkeys that were newly trained for the experiment. Monkeys in faded colors (next to the arrows) indicate those used in the previous experiment. (**b**) Extent of lesions in the three monkeys with bilateral lesions to ventral striatum (VS) who performed the What-only and What/Where task. Adapted from Rothenhoefer, K. M. et al. Effects of Ventral Striatum Lesions on Stimulus-Based versus Action-Based Reinforcement Learning. Journal of Neuroscience 19 July 2017, 37 (29) 6902-6914; DOI: 10.1523/JNEUROSCI.0631-17.2017. (**c**) Extent of lesions in the four monkeys with bilateral lesions to amygdala who performed the What-only task. This article was published in Neuron Volume 92, Costa VD, Dal Monte O, Lucas DR, Murray EA, Averbeck BB, “Amygdala and Ventral Striatum Make Distinct Contributions to Reinforcement Learning,” 505-517, Copyright Elsevier (2016). (**d**) Extent of lesions in the four monkeys with bilateral lesions to amygdala who performed the What/Where task. Used with permission of Oxford University Press, from “Effects of Amygdala Lesions on Object-Based Versus Action-Based Learning in Macaques,” Taswell CA, Costa VD, Basile BM, Pujara MS, Jones B, Manem N, Murray EA, Averbeck BB, *31*(1), 2021; permission conveyed through Copyright Clearance Center, Inc.

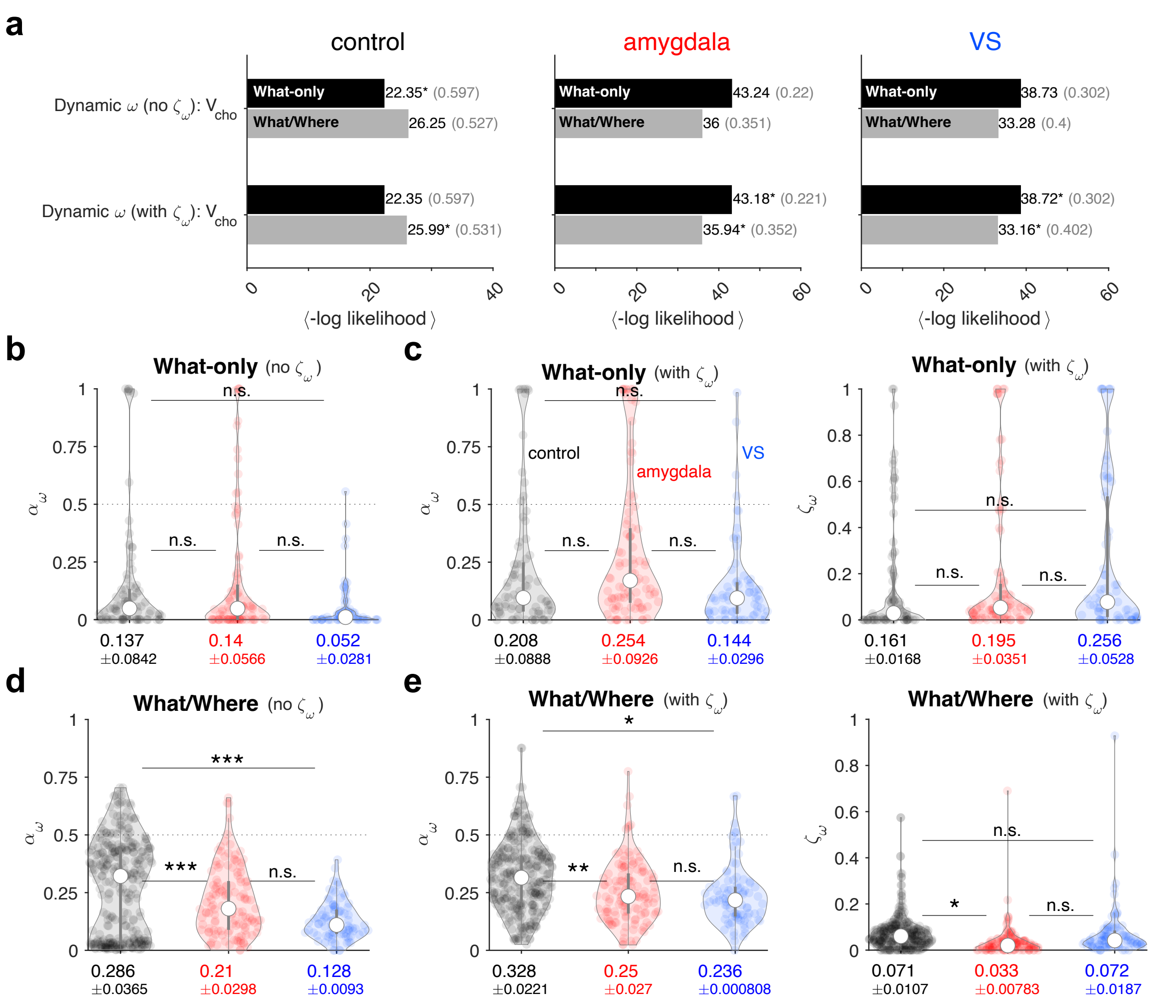

Supplementary Figure 18. Comparison between dynamic models with or without passive decay in arbitration weight, and estimated parameters in the better-fitting model. (**a**) Goodness of fit using five-fold cross-validation of dynamic models with or without passive decay for ω. Numbers in parenthesis indicate McFadden R^2 (Eq. 20). Model with the additional passive decay mechanism better accounts for the choice behavior in all groups, especially during the What/Where task. (**b**) Distribution of estimated parameters for sessions in controls (black), amygdala (red), and VS (blue) groups during the What-only task, showing parameters for α_ω in the above model without passive decay in ω. Asterisks indicate significant effects of group difference (mixed-effects analysis with random effects of subjects, *parameter* ~ *group* + (1+*sess_perc*|*subject*)); *: $p < .05$, **: $p < .01$, ***: $p < .001$; n=243 sessions). Circles in the violin plots indicate medians, and the numbers on the X-axis indicate mean±SEM across subjects. (**c**) Same plots as in (b) but for the above model with passive decay in ω, showing parameters for α_ω (model arbitration/update rate, left) and ζ_ω (decay rate for ω, right). Arbitration rates α_ω tended to be larger than the passive decay rate ζ_ω in control and amygdala groups (mixed-effects analysis on paired difference, $\alpha_\omega - \zeta_\omega$ ~ 1 + (1+*sess_perc*|*subject*)); controls: $\beta_0 = 0.0497$, $p = .515$; amygdala: $\beta_0 = 0.067$, $p = .459$; VS: $\beta_0 = -0.0929$, $p = .112$). (**d**) Same plot as in (b) but for the What/Where task, in the model without passive decay in ω (n=532 sessions). (**e**) Same plots as in (c) but for What/Where task, in the model with passive decay in ω. Arbitration rates α_ω were significantly larger than the passive decay rate ζ_ω across all groups, suggesting that α_ω is the primary source of the transitions in ω (mixed-effects analysis on $\alpha_\omega - \zeta_\omega$; controls: $\beta_0 = 0.269$, $p = 2.18\times10^{-25}$; amygdala: $\beta_0 = 0.215$, $p = 3.02\times10^{-24}$; VS: $\beta_0 = 0.163$, $p = 1.93\times10^{-16}$). Source data are provided as a Source Data file.

Supplementary Tables

Supplementary Tables 1–14. Summary of the main mixed-effects regression analyses. Reported below are the list of regression models, along with their hypotheses and corresponding results. All reported p-values are the exact values (two-sided) obtained before applying any correction for multiple comparisons. Shaded gray color indicates statistics relevant to each hypothesis reported in the text. Control monkeys were selected as the reference group wherever group comparison has been made. For the *blockType* variable in the What/Where task, 'What' block was used as the reference category. For the random effects, we included subject-level intercept and random slopes for the long-term adjustment effect, across the entire experiment and within each session. The variable *sess_perc* represents the proportion of the sessions completed and was mean-centered within each subject. Similarly, *block_in_sess* refers to the block number within a session and was also mean-centered within each subject. Source data are provided as a Source Data file.

Supplementary Table 1. Comparison of performance across the three groups during the What-only task.

**Model**	*Block-wise P(Better) ~ group + (1 + sess_perc + block_in_sess |subject)*				
**Hypothesis**	Performance is significantly reduced in the brain-lesioned group.				
**Coeff.**	**Estimate**	**SE**	**t-stat**	**DF**	**P-value**
Intercept	0.7567	0.012535	60.367	4099	0
group_amygdala	-0.17156	0.017763	-9.6585	4099	7.7214e-22
group_VS	-0.095504	0.019631	-4.8651	4099	1.1871e-06
**Planned contrasts**	Difference between amygdala- and VS-lesioned groups				
group_amyg – group_VS	-0.076055	0.019662	-3.868	4099	0.00011142

Supplementary Table 2. Comparison of performance across the three groups during the What/Where task.

**Model**	*Block-wise P(Better) ~ group*blockType + (1+ blockType + sess_perc + block_in_sess |subject)*				
**Hypothesis**	Performance is significantly reduced in the brain-lesioned group.				
**Coeff.**	**Estimate**	**SE**	**t-stat**	**DF**	**P-value**
Intercept	0.74626	0.022347	33.394	9498	2.7758e-231
group_amyg	-0.10516	0.03582	-2.9357	9498	0.0033362
group_VS	-0.20892	0.039254	-5.3222	9498	1.0484e-07
blockType_Where	-0.025193	0.011474	-2.1958	9498	0.028134
group_amyg: blockType_Where	0.034786	0.021032	1.6539	9498	0.098172
group_VS: blockType_Where	0.1717	0.02165	7.9306	9498	2.4265e-15
**Planned contrasts**	Group differences during What or Where blocks				
group_amyg – group_VS	0.10376 (amyg – VS)	0.042722	2.4288	9498	0.015167
group_amyg + group_amyg: blockType_Where	-0.07037 (amyg - control)	0.029571	-2.3797	9498	0.017346
group_VS + group_VS: blockType_Where	-0.037222 (VS - control)	0.031844	-1.1689	9498	0.24248
(group_amyg + amyg:Where) – (group_VS + group_VS:Where)	-0.033148 (amyg - VS)	0.03556	-0.93216	9498	0.35128

Supplementary Table 3. Comparison of effective arbitration rates ($\Delta\psi = \psi_+ - \psi_-$) across the three groups during the What-only task.

**Model**	*Block-wise Δψ ~ 1 + group + (1 + sess_perc + block_in_sess |subject)*				
**Hypothesis**	Arbitration rates toward stimulus system are significantly larger than those toward action system in controls, and this effect is reduced in amygdala-lesioned monkeys				
**Coeff.**	**Estimate**	**SE**	**t-stat**	**DF**	**P-value**
Intercept	0.092809	0.004193	22.134	4099	1.2391e-102
group_amygdala	-0.078571	0.006064	-12.958	4099	1.1508e-37
group_VS	-0.070159	0.007225	-9.7103	4099	4.7004e-22
**Planned contrasts**	Mean of Δψ in brain lesioned groups				
Intercept + group_amyg	0.014238 (amyg mean)	0.0043799	3.2507	4099	0.0011606
Intercept + group_VS	0.022649 (VS mean)	0.005884	3.8493	4099	0.00012028
group_amyg – group_VS	-0.0084115	0.0073352	-1.1467	4099	0.25156

Supplementary Table 4. Comparison of effective arbitration rates across the three groups during the What/Where task.

**Model**	*Block-wise Δψ ~ group*blockType + (1+ blockType + sess_perc + block_in_sess |subject)*				
**Hypothesis**	Effective arbitration rates toward the correct system (for a given block) are appropriately biased in controls, but amygdala group is not differentiated in the two rates.				
**Coeff.**	**Estimate**	**SE**	**t-stat**	**DF**	**P-value**
Intercept	0.044358	0.007343	6.0409	9498	1.5905e-09
group_amyg	-0.032996	0.011793	-2.798	9498	0.0051522
group_VS	-0.097925	0.012858	-7.6156	9498	2.8745e-14
blockType_Where	-0.07444	0.016061	-4.6347	9498	3.6214e-06
group_amyg: blockType_Where	0.05961	0.026653	2.2365	9498	0.025341
group_VS: blockType_Where	0.054152	0.0286	1.8934	9498	0.058337
**Planned contrasts**	Mean of Δψ during Where blocks in controls,<br>Mean of Δψ during each block type in amygdala group				
Intercept + blockType_Where	-0.030082 (Control, Where)	0.011626	-2.5874	9498	0.0096838
Intercept + group_amyg	0.011362 (Amyg, What)	0.0092275	1.2314	9498	0.21822
Intercept + group_amyg + blockType_Where+group_amyg: blockType_Where	-0.003467 (Amyg, Where)	0.015619	-0.22199	9498	0.82432
Other Post-hoc contrasts	-0.053566 (VS, What)	0.010555	-5.0748	9498	3.9533e-07
	-0.073854 (VS, Where)	0.017323	-4.2633	9498	2.034e-05

Supplementary Table 5. Comparison of differentiation in two arbitration rates across the three groups during the What/Where task.

**Model**	*Block-wise |$\Delta\psi$| ~ group + (1 + sess_perc + block_in_sess |subject)*				
**Hypothesis**	Amygdala group shows minimal differentiation between the two rates.				
**Coeff.**	**Estimate**	**SE**	**t-stat**	**DF**	**P-value**
Intercept	0.055852	0.006268	8.9114	9501	5.9665e-19
group_amygdala	-0.025358	0.010026	-2.5293	9501	0.011445
group_VS	0.0028819	0.010941	0.26341	9501	0.79224
**Planned contrasts**	Difference between amygdala and VS groups				
group_amyg – group_VS	-0.02824	0.011902	-2.3727	9501	0.017676

Supplementary Table 6. Comparison of relative sensitivity to stimulus and action value signals ($\Delta\beta = \beta_{\text{stim}} - \beta_{\text{action}}$) across the three groups during the What-only task (**Figure 4b** inset).

**Model**	*Session-wise $\Delta\beta$ ~ group + (1 + sess_perc |subject)*				
**Hypothesis**	Relative sensitivity to stimulus is not different between control and amygdala group, but it is reduced in VS group.				
**Coeff.**	**Estimate**	**SE**	**t-stat**	**DF**	**P-value**
Intercept	4.9348	1.7631	2.799	240	0.0055428
group_amygdala	-2.6925	2.4859	-1.0831	240	0.27984
group_VS	-7.4768	2.7272	-2.7415	240	0.0065759
**Planned contrasts**	Difference between amygdala and VS groups				
group_amyg – group_VS	4.7843	2.7204	1.7587	240	0.079906

Supplementary Table 7 Comparison of relative sensitivity to stimulus and action value signals ($\Delta\beta = \beta_{\text{stim}} - \beta_{\text{action}}$) across the three groups during the What/Where task (**Figure 4c** inset).

**Model**	*Session-wise $\Delta\beta$ ~ group + (1 + sess_perc |subject)*				
**Hypothesis**	Relative sensitivity is not different between control and amygdala group, but it is reduced in VS group.				
**Coeff.**	**Estimate**	**SE**	**t-stat**	**DF**	**P-value**
Intercept	2.3268	0.95107	2.4465	529	0.014749
group_amygdala	0.54218	1.5067	0.35984	529	0.71911
group_VS	-3.53	1.6544	-2.1337	529	0.033323
**Planned contrasts**	Difference between amygdala and VS groups				
group_amyg – group_VS	4.0722	1.7883	2.2771	529	0.02318

Supplementary Table 8 Comparison of initial effective arbitration weight (Ω_0) across the three groups during the What/Where task.

**Model**	*Session-wise Ω_0 ~ group + (1 + sess_perc |subject)*				
**Hypothesis**	Initial "effective" arbitration weight is not different between amygdala and VS groups.				
**Coeff.**	**Estimate**	**SE**	**t-stat**	**DF**	**P-value**
Intercept	0.44977	0.028853	15.589	529	2.3315e-45
group_amygdala	-0.20808	0.045898	-4.5336	529	7.1792e-06
group_VS	-0.23227	0.050245	-4.6228	529	4.7636e-06
**Planned contrasts**	Difference between amygdala and VS groups				
group_amyg – group_VS	0.024192	0.054463	0.44419	529	0.65709

Supplementary Table 9 Comparison of initial arbitration weight (ω_0) across the three groups during the What/Where task.

**Model**	*Session-wise ω_0 ~ group + (1 + sess_perc |subject)*				
**Hypothesis**	Initial "effective" arbitration weight is not different between amygdala and VS groups.				
**Coeff.**	**Estimate**	**SE**	**t-stat**	**DF**	**P-value**
Intercept	0.38545	0.042547	9.0594	529	2.53e-18
group_amygdala	-0.19421	0.067522	-2.8762	529	0.0041873
group_VS	-0.11905	0.073843	-1.6122	529	0.10751
**Planned contrasts**	Difference between amygdala and VS groups				
group_amyg – group_VS	-0.075155	0.079947	-0.94005	529	0.34762

Supplementary Table 10 Comparison of the changes in the initial arbitration weight (ω_0) across the three groups (after scaling by ρ), during the What/Where task.

**Model**	*Session-wise (Ω_0 - ω_0) ~ group + (1 + sess_perc |subject)*				
**Hypothesis**	Changes to effective arbitration weight after re-scaling is larger in amygdala group compared to VS.				
**Coeff.**	**Estimate**	**SE**	**t-stat**	**DF**	**P-value**
Intercept	0.062704	0.025184	2.4898	529	0.013087
group_amygdala	-0.012829	0.040057	-0.32027	529	0.74889
group_VS	-0.12382	0.044075	-2.8094	529	0.0051467
**Planned contrasts**	Difference between amygdala and VS groups				
group_amyg – group_VS	0.111	0.047735	2.3252	529	0.020437

Supplementary Table 11. Long-term adjustment in stimulus-based strategy ($ERDS_{Stim}$) during the What-only task.

**Model**	*Block-wise $ERDS_{Stim}$ ~ group* session_perc + (1+ session_perc + block_in_sess |subject)*				
**Hypothesis**	There is significant effect of long-term adjustment in stimulus-based strategy				
**Coeff.**	**Estimate**	**SE**	**t-stat**	**DF**	**P-value**
Intercept	0.46524	0.03119	14.916	4096	4.9007e-49
group_amyg	0.38501	0.044163	8.718	4096	4.0496e-18
group_VS	0.21605	0.048054	4.496	4096	7.1158e-06
session_perc	-0.076933	0.045091	-1.7062	4096	0.088053
group_amyg: session_perc	0.043531	0.065166	0.668	4096	0.50417
group_VS: session_perc	-0.54922	0.080032	-6.8625	4096	7.7832e-12
**Planned contrasts**	Slope for each brain-lesioned group<br>Difference in slope between amygdala and VS group				
session_perc + group_amyg: session_perc	-0.033402 (amyg slope)	0.047047	-0.70997	4096	0.47776
session_perc + group_VS: session_perc	-0.62615 (VS slope)	0.06612	-9.4699	4096	0
group_amyg: session_perc – group_VS: session_perc	0.59275 (amyg vs. VS)	0.081149	7.3044	4096	3.3307e-13

Supplementary Table 12. Long-term adjustment in action-based strategy ($ERDS_{Action}$) during the What-only task.

Model	*Block-wise $ERDS_{Act}$ ~ group* session_perc + (1+ session_perc + block_in_sess |subject)*				
**Hypothesis**	There is significant effect of long-term adjustment in action-based strategy				
**Coeff.**	**Estimate**	**SE**	**t-stat**	**DF**	**P-value**
Intercept	0.98149	0.005686	172.62	4096	0
group_amyg	-0.058954	0.009191	-6.4141	4096	1.5771e-10
group_VS	-0.016861	0.013944	-1.2092	4096	0.22666
session_perc	-0.019254	0.042687	-0.45106	4096	0.65197
group_amyg: session_perc	0.033012	0.06049	0.54574	4096	0.58527
group_VS: session_perc	0.21104	0.068511	3.0803	4096	0.002081
**Planned contrasts**	Slope for each brain-lesioned group,<br>Difference in slope between amygdala and VS group				
session_perc + group_amyg: session_perc	0.013757 (amyg slope)	0.042858	0.321	4096	0.748227
session_perc + group_VS: session_perc	0.191783 (VS slope)	0.053587	3.578898	4096	0.000349
group_amyg: session_perc – group_VS: session_perc	-0.178026 (amyg vs. VS)	0.068618	-2.594448	4096	0.009508

Supplementary Table 13. Long-term adjustment in reaction time (RT) during the What-only task.

Model	*Block-wise median RT ~ group*session_perc + (1+ session_perc + block_in_sess |subject)*				
**Hypothesis**	There is significant effect of long-term adjustment in action-based strategy				
**Coeff.**	**Estimate**	**SE**	**t-stat**	**DF**	**P-value**
Intercept	223.6	15.517	14.41	4096	5.8459e-46
group_amyg	28.694	22.016	1.3033	4096	0.19254
group_VS	-33.208	24.017	-1.3827	4096	0.16684
session_perc	6.4037	4.7777	1.3403	4096	0.18022
group_amyg: session_perc	-5.3131	6.7961	-0.78179	4096	0.43439
group_VS: session_perc	24.258	7.9722	3.0429	4096	0.0023581
**Planned contrasts**	Slope for each brain-lesioned group,<br>Difference in slope between amygdala and VS group				
session_perc + group_amyg: session_perc	1.0906 (amyg slope)	4.833257	0.225644	4096	0.82149
session_perc + group_VS: session_perc	30.662 (VS slope)	6.381877	4.804542	4096	1.6066e-06
group_amyg: session_perc – group_VS: session_perc	-29.5714 (amyg vs. VS)	8.005544	-3.693865	4096	2.2375e-04

Supplementary Table 14. Long-term adjustment in the initial effective arbitration weight (Ω_0) during the What-only task.

**Model**	*Session-wise Ω_0 ~ group*session_perc + (1+ session_perc |subject)*				
**Hypothesis**	There is significant effect of long-term adjustment in action-based strategy				
**Coeff.**	**Estimate**	**SE**	**t-stat**	**DF**	**P-value**
Intercept	0.89228	0.069903	12.764	237	9.5239e-29
group_amyg	-0.263	0.09874	-2.6635	237	0.008263
group_VS	-0.12909	0.10846	-1.1902	237	0.23517
session_perc	-0.11273	0.061507	-1.8328	237	0.068085
group_amyg: session_perc	0.13703	0.084658	1.6186	237	0.10686
group_VS: session_perc	0.56793	0.13812	4.1117	237	5.4173e-05
**Planned contrasts**	Slope for each brain-lesioned group<br>Difference in slope between amygdala and VS group				
session_perc + group_amyg: session_perc	0.024298<br>(amyg slope)	0.058171	0.41771	4096	0.67654
session_perc + group_VS: session_perc	0.4552<br>(VS slope)	0.12367	3.6806	4096	2.8809e-04
group_amyg: session_perc – group_VS: session_perc	-0.4309<br>(amyg vs. VS)	0.13667	-3.1528	4096	0.0018258

Supplementary Table 15. Reinforcement learning models used to fit choice data, description of parameters, and their fitting ranges. Each row provides a short description of a given RL model, its parameters, and the range of values used for fitting to each session. Note that the first five parameters are common across all models.

Model	Model description (# of parameters)	Description of parameters and their fitting ranges
$RL_{Stim\text{-}only}$	Single-system RL model that learns stimulus values only. (5)	(1) α_+ : learning rate for rewarded trials, [0 1]<br>(2) α_- : learning rate for unrewarded trials, [0 1]<br>(3) β_0 : side bias, positive if preferring right option, [-1 1]<br>(4) β_1 : inverse temperature, [1 100]<br>(5) ζ : decay or forgetting rate for the unchosen option, [0 1]
$RL_{Action\text{-}only}$	Single-system RL model that learns action values only. (5)	Parameters (1)−(5): same as the first model.
Static ω ($RL_{Stim+Act}$)	Two-system model with a fixed weighting between stimulus and action values. (6)	Parameters (1)−(5): same as the first model.<br><br>(6) ω_{static}: fixed arbitration weight
Dynamic ω: |RPE|	Two-system model with dynamic weighting, with reliability based on |RPE|. (8)	Parameters (1)−(5): same as the first model.<br>(6) ω_0: initial arbitration weight, [0 1]<br>(7) α_ω : update rate for arbitration weight, [0 1]<br>(8) ζ_ω : passive decay rate for arbitration weight, [0 1]
Dynamic ω: V_{cho}	Two-system model with dynamic weighting, with reliability based on V_{Chosen}. (8)	Parameters (1)−(5): same as the first model.<br>(6) ω_0: initial arbitration weight, [0 1]<br>(7) α_ω : update rate for arbitration weight, [0 1]<br>(8) ζ_ω : passive decay rate for arbitration weight, [0 1]
Dynamic ω-ρ (V_{cho})	Two-system models with dynamic weighting (with reliability based on V_{Chosen}) and separate baseline signals for stimulus- and action-based learning. (9)	Parameters (1)−(5): same as the first model.<br>(6) ω_0: initial arbitration weight, [0 1]<br>(7) α_ω : update rate for arbitration weight, [0 1]<br>(8) ζ_ω : passive decay rate for arbitration weight, [0 1]<br>(9) ρ: a hyperparameter, estimated for each subject, measuring the baseline ratio of signals from the stimulus-based to that from the action-based system, [0 1]

Supplementary Table 16. Cross-validated negative log-likelihoods of RL models, separately for each monkey in a given task. Reported are the mean negative log-likelihoods for each monkey across all tested block instances (five-fold cross-validation), rounded to the nearest hundredth. Asterisks indicate the model with the lowest value of negative log-likelihood for each task. The initial and the number in parenthesis indicate a unique identifier for each monkey, as shown in **Supplementary Figure 17**. Source data are provided as a Source Data file.

What-only task (11 monkeys)

Groups	**Controls**				**Amygdala-lesioned**				**VS-lesioned**		
Subjects	E (1)	G (2)	M (3)	N (4)	C (5)	CB (6)	P (7)	Q (8)	F (9)	A (10)	G (11)
RL$_{\text{Stim-only}}$	25.77	16.34	24.92	25.86	49.24	39.31	43.41	42.85	43.04	42.41	44.53
RL$_{\text{Action-only}}$	54.46	55.18	54.67	54.96	50.14	45.76	50.36	54.51	48.94	48.95	48.95
Static ω (RL$_{\text{Stim+Act}}$)	25.65	16.05	24.81	25.77	47.86	37.79	42.44	42.42	37.62	38.63	43.8
Dynamic ω: |RPE|	25.63	16.06	24.81	25.77	47.73	37.67	42.36	42.40	37.35	38.24	43.8
Dynamic ω: V$_{\text{cho}}$	25.39	14.97	24.66	25.69	47.63	37.46	42.15	42.13	36.24	37.69	43.74
Dynamic ω-ρ (V$_{\text{cho}}$)	25.38 *	14.96 *	24.55 *	25.65 *	47.49 *	37.39 *	42.15 *	42.13 *	36.24 *	37.53 *	43.73 *

What/Where task (13 monkeys)

Groups	**Controls**						**Amygdala-lesioned**				**VS-lesioned**		
Subjects	G (2)	R (12)	U (13)	V (13)	W (14)	B (16)	Be (17)	En (18)	MJ (19)	Z (20)	F (9)	A (10)	G (11)
RL$_{\text{Stim-only}}$	38.27	41.21	49.24	42.2	44.92	42.48	53.16	47.25	45.47	51.27	54.27	53.48	52.67
RL$_{\text{Action-only}}$	47.76	47.3	43.89	43.42	41.36	40.33	36.83	45.32	47.27	47.73	34.92	33.69	36.83
Static ω (RL$_{\text{Stim+Act}}$)	26.07	30.16	35.88	25.65	26.26	22.59	33.37	37.08	35.51	43.67	33.6	32.9	35.36
Dynamic ω: |RPE|	25.92	30.09	35.76	24.62	25.25	21.68	32.38	36.73	33.32	43.47	33.44	32.67	34.93
Dynamic ω: V$_{\text{cho}}$	24.92	29.32	34.72	22.88	23.31	18.53	31.88	35.86	33.44	42.57	32.68	32.23	34.43
Dynamic ω-ρ (V$_{\text{cho}}$)	24.84 *	29.19 *	34.56 *	22.88 *	23.29 *	18.51 *	31.86 *	35.66 *	33.33 *	42.41 *	32.62 *	32.18 *	34.43 *
